# Generation and Characterization of an Inducible Cx43 Overexpression System in Mouse Embryonic Stem Cells

**DOI:** 10.3390/cells11040694

**Published:** 2022-02-16

**Authors:** Pia Niemann, Miriam Schiffer, Daniela Malan, Sabine Grünberg, Wilhelm Roell, Caroline Geisen, Bernd K. Fleischmann

**Affiliations:** 1 Institute of Physiology I, Life and Brain Center, Medical Faculty, University of Bonn, 53127 Bonn, Germany; s6piniem@uni-bonn.de (P.N.); mschiffer@uni-bonn.de (M.S.); dmalan@uni-bonn.de (D.M.); sabine.gruenberg@uni-bonn.de (S.G.); 2 Department of Cardiac Surgery, University Hospital Bonn, 53127 Bonn, Germany; wroell@uni-bonn.de

**Keywords:** Cx43 overexpression system, functional Cx43 gap junctions, embryonic stem cells, AAV2.1-Cre-mediated induction, P2A, mCherry expression

## Abstract

Connexins (Cx) are a large family of membrane proteins that can form intercellular connections, so-called gap junctions between adjacent cells. Cx43 is widely expressed in mammals and has a variety of different functions, such as the propagation of electrical conduction in the cardiac ventricle. Despite Cx43 knockout models, many questions regarding the biology of Cx43 in health and disease remain unanswered. Herein we report the establishment of a Cre-inducible Cx43 overexpression system in murine embryonic stem (ES) cells. This enables the investigation of the impact of Cx43 overexpression in somatic cells. We utilized a double reporter system to label Cx43-overexpressing cells via mCherry fluorescence and exogenous Cx43 via fusion with P2A peptide to visualize its distribution pattern. We proved the functionality of our systems in ES cells, HeLa cells, and 3T3-fibroblasts and demonstrated the formation of functional gap junctions based on dye diffusion and FRAP experiments. In addition, Cx43-overexpressing ES cells could be differentiated into viable cardiomyocytes, as shown by the formation of cross striation and spontaneous beating. Analysis revealed faster and more rhythmic beating of Cx43-overexpressing cell clusters. Thus, our Cx43 overexpression systems enable the investigation of Cx43 biology and function in cardiomyocytes and other somatic cells.

## 1. Introduction

Connexins (Cx) are a large family of transmembrane proteins that are primarily localized in the plasma membrane, but also in intracellular organelles, such as mitochondria [1,2]. In mice, 20 different isotypes of Cx have been identified [3]; they are widely expressed in all organs and tissues starting from early embryonic development [4]. All Cx share the same structural features consisting of an amino- and carboxy-terminus located in the cytosol, one intracellular loop and four transmembrane domains. Six Cx form a connexon, and two adjacent connexons in the plasma membrane of neighboring cells can form functional intercellular channels, the gap junctions [5]. Numerous gap junctions form clusters, called plaques [6]. Cx biology is complex, as different Cx isotypes can assemble and form heteromeric connexons and/or even heterotypic gap junctions [7]. More recent work proposes the existence of hemichannels, which are thought to open under pathophysiological conditions [8].

Gap junctions are known to have pleiotropic functions, such as the exchange of nutrients, signaling components, and ions below a MW of 1.5 kDa. Therefore, these proteins mediate electrical and metabolic cell-to-cell coupling. This explains their vital role and the early embryonic lethality upon knocking out specific Cx genes, e.g., Cx45 [9]. In addition, germ line and even somatic mutations of Cx encoding genes can result in various diseases. In fact, mutations in 10 different human Cx have been linked to 28 distinct genetic disorders, e.g., Cx26-related deafness [10], Cx46-related cataracts, and Cx32-related X-linked Charcot–Marie–Tooth neuropathy [11].

One of the most widely distributed and studied members of the Cx family is Cx43, which is known to be strongly expressed in the heart [12]. The heart forms an electrical syncytium, and the electrical conduction is critically dependent on the expression and function of Cx43 in cardiomyocytes of the atrium and ventricle, whereas Cx40 is preferentially expressed in atrial cardiomyocytes and Cx45 in cells of the sinus and AV node [13]. In the healthy human heart, Cx43 gap junctions are primarily located at intercalated discs connecting two neighboring cardiomyocytes providing orderly propagation of the electrical conduction throughout the working myocardium. This critical role of gap junctions for heart function is exemplified by mutations of Cx, which are strongly expressed in the heart, causing diseases such as Cx40-related atrial fibrillation [14] and Cx43-related oculodentodigital dysplasia [15]. Altered expression, distribution, and modulation of Cx43 channels and/or gap junctions are thought to occur in pathophysiological situations and to be potentially involved in the generation of life-threatening ventricular arrhythmias. In addition, myocardial ischemia and infarction have been reported to result in an altered distribution pattern of Cx43, e.g., atypical cellular localization in the lateral cardiomyocyte membrane, named lateralization [16,17]; the pathophysiological significance is still under debate [18,19,20]. Furthermore, hypoxia and high cytosolic Ca^2+^ have been reported to change cardiac Cx43 gap junction gating and, consequently, electrical conduction properties [21].

Given the lack of highly specific pharmacological agonists and blockers of Cx, small Cx43 mimetic peptides with either blocking (e.g., Gap26, Gap27) or agonist functions (Gap134, αCT1) have been developed and tested for the targeting of heart disease [22]. Besides specificity and efficacy issues, the in vivo delivery of these compounds needs to be further developed [23,24,25]. Most of the actual mechanistic understanding of Cx43 function for heart development and disease has been obtained from knockout (KO) mouse models [21]. These have revealed that null mutations of the Cx43 gene result in morphogenic malformations of the outflow tract of the heart and perinatal death because of asphyxia [26]. This phenotype was primarily caused by Cx43-related defects of neural crest cells [27]. Cell type-specific and inducible deletion of Cx43 in cardiomyocytes resulted in severe ventricular arrhythmias, underscoring its critical role in propagating electrical conduction in the heart. These studies also revealed that only 5–10% of Cx43 protein is required to maintain its normal function [28]. However, many aspects of the biology and function of Cx43 in cardiomyocytes and in the heart are still poorly understood, in particular Cx43’s contribution to cardiomyocyte development, its effects in disease and malfunctioning, the relevance of its lateralization, and the formation and functional significance of hemichannels. To address these aspects, we have taken a different approach from KOs by generating an inducible Cx43 overexpression model in pluripotent mouse embryonic stem (ES) cells. This strategy was chosen, since earlier experiments in mice illustrated that ubiquitous overexpression of Cx43 resulted in embryonic and postnatal morbidity and reduced postnatal viability [29]. We reasoned that Cx43 overexpression could also have (adverse) effects on the propagation, development, and differentiation of native cells and/or ES cell-derived cell populations in vitro. Therefore, we considered it a clear advantage to be able to induce Cx43 overexpression at later stages of the in vitro differentiation of (ES) cells.

## 2. Materials and Methods

### 2.1. Generation of the CAG-floxSTOP-Cx43-P2A-mCherry-Ai6 Construct

The sequence of the mouse Cx43 cDNA, followed in frame by the DNA sequence of the GSG-P2A peptide and the mCherry cDNA, was synthesized together with flanking FseI sites by GeneArt Gene Synthesis (Thermo Fisher Scientific, Waltham, MA, USA) and subcloned into the pMK-RQ standard vector from Thermo Fisher. The customized 1968 bp DNA fragment was cut out by digestion with FseI, isolated and ligated downstream of the CAG promoter and a floxed stop cassette into the FseI sites of the Ai6 vector (Addgene, Watertown, MA, USA, #22798) [30], thereby exchanging the zsGreen reporter. The Ai6 vector is a Rosa26 (Rs26) targeting vector that allows homologous integration of expression and NeoR/KanR cassettes into the mouse Rs26 locus by co-transfection with specific zinc finger nucleases. The Ai6 vector contains the post-transcriptional regulatory element of woodchuck hepatitis virus (WPRE) to increase transgene expression [31]. All DNAs were prepared using the EndoFree Plasmid Maxi Kit from Qiagen (Venlo, The Netherlands). To verify correct cloning of the CAG-floxSTOP-Cx43-P2A-mCherry-Ai6 construct, restriction analyses and sequencing (Eurofins Genomics, Ebersberg, Germany) were performed. For molecular cloning procedures and sequence analyses, SnapGene Viewer 5.3.2 (from Insightful Science, available at snapgene.com, San Diego, CA, USA) and Clone Manager Professional 9 (Sci Ed Software LLC, Denver, CO, USA) were used.

### 2.2. Cell Culture

Undifferentiated murine G4 hybrid embryonic stem (ES) cells [32] were cultivated in KnockOut Dulbecco’s modified Eagle’s medium (DMEM high glucose, Gibco, Thermo Fisher Scientific, supplemented with 15% *v*/*v* fetal calf serum (FCS, PAN Biotech, Aidenbach, Germany), 0.1 mM nonessential amino acids (NEAA, Gibco, Thermo Fisher Scientific), 2.4 mM L-glutamine (Thermo Scientific), 100 U/mL/100 µg/mL penicillin/streptomycin (P/S), 500 U/mL leukemia inhibitory factor (LIF), and 0.1 mM β-mercaptoethanol (β-ME, Gibco, Thermo Fisher Scientific)) in tissue culture flasks coated with irradiated neomycin-resistant mouse embryonic fibroblasts (PMEF-NL, Merck, Darmstadt, Germany). ES cells were differentiated using embryoid body (EB) formation via hanging drop method [33]. For differentiation, cells were harvested and resuspended in Iscove’s MEM high-glucose medium (IMDM, Thermo Fisher) supplemented with 20% *v*/*v* FCS, 100 U/mL/100 µg/mL P/S, 0.1 mM NEAA, and 0.1 mM β-ME in the absence of LIF. Irradiated fibroblasts were reduced by differential sedimentation, and the ES cell-enriched suspension was dripped (400 cells per 20 µL drop) on the inner side of the lid of a cell culture dish. After two days of incubation, the hanging drops were rinsed off with 20% IMDM and further differentiated in suspension culture on a shaking plate (horizontal shaker, LAUDA-GFL, Burgwedel, Germany).

HPV18-positive HeLa (RRID: CVCL_0030) human cervical carcinoma cells were maintained in DMEM (pH 7.2), plus additives (20% *v*/*v* FCS, 120 U/mL/120 µg/mL P/S, 0.1 mM NEAA, 2 mM L-glutamine, 1.2 mM sodium pyruvate (Gibco, Thermo Fisher Scientific), and 0.12 mM β-ME).

NIH/3T3 mouse fibroblasts (3T3, ATCC, Manassas, VA, USA, #CRL1658) were cultivated in DMEM (plus 10% *v*/*v* FCS, 100 U/mL/100 µg/mL P/S, 0.1 mM NEAA, and 0.1 mM β-ME).

All cells were cultivated at 37 °C and 5% CO_2_.

### 2.3. CAG-floxSTOP-Cx43-P2A-mCherry-Ai6 Transfection

For the generation of transgenic cells, either mouse G4 ES cells (5.4 × 10^6^) or 3T3 mouse fibroblasts (4 × 10^6^) were mixed in 800 µL PBS with 30 µg circular CAG-floxSTOP-Cx43-P2A-mCherry-Ai6 plasmid DNA and 10 µg zinc finger nucleases pCMV-rosaR4-KKR (Addgene, #37199) and pCMV-rosaL6-ELD mutations (Addgene, #37198) and electroporated (ES cells: 240 V, 500 µF, 1 pulse; 3T3-fibroblasts: 180 V, 950 µF, 1 pulse) using the Gene Pulser Xcell Electroporation Systems (Bio-Rad, Hercules, CA, USA). Two days after electroporation, transgenic G4 ES cells and 3T3 cells were selected by adding 225 µg/mL and 800 µg/mL G418 (neomycin, Gibco, Thermo Fisher Scientific), respectively, to the medium for ten (G4 ES cells) or twelve days (3T3 mouse fibroblasts).

HeLa cells (60% confluency) were transfected with the PacI digested linearized CAG-floxSTOP-Cx43-P2A-mCherry-Ai6 construct using the TransIT-HeLaMONSTER Transfection Kit (Mirus Bio LLC, Madison, WI, USA) according to the manufacturer’s instructions. Two days after transfection, transgenic HeLa cells were selected by adding 600 µg/mL G418 to the medium for twelve days.

Resistant ES cells and HeLa colonies were isolated and expanded in 24-well plates; in case of the ES cells, these were coated with irradiated neomycin-resistant fibroblasts. Colonies of resistant transgenic 3T3 cells were pooled, and a single cell dilution of 3T3-fibroblasts was performed, as depicted in Figure 3a.

Neomycin-resistant colonies were analyzed for the presence of the expression cassette by PCR (see 2.9) and mCherry fluorescence after AAV2.1-Cre transduction.

### 2.4. Generation of Transgene-Expressing Cre^+^ Cell Lines

The AAV2.1-Cre virus (pENN.AAV.CMVs.PI.Cre.rBG, Addgene, #105537-AAV1, 1.2 × 10^13^ GC/mL) was titrated on a G4 ES cell line (BAG3P209L-eGFPflox3) [34] expressing eGFP after Cre induction; 5.6 × 10^10^ genome copies/mL per 7500 cells were used. Viral transduction was performed in cell culture medium of CAG-floxSTOP-Cx43-P2A-mCherry-Ai6 transfected (Cre^−^) lines of ES, HeLa cells, and 3T3-fibroblasts. Two days post-transduction, the virus was washed off, and at day three post-transduction, mCherry-expressing colonies were observed. These were either picked, or single cell dilutions were performed.

For immunostainings, cells were cultured on fibronectin (0.5 µg/mL, Sigma-Aldrich, Merck) or 0.1% gelatin- (Merck) coated (I) 96 well glass-bottom plates (Greiner, Kremsmünster, Austria) or (II) cover glasses (VWR, Radnor, PA, USA), fixed with 4% Formaldehyde (FA) (PanReac AppliChem, Darmstadt, Germany) for 30 min.

### 2.5. Generation of AAV2.6-Cx43-P2A-mCherry Virus

The Cx43-P2A-mCherry insert was directly cloned under control of the CAG promoter of the pAAV.CAG.hChR2(H134R)-mCherry.WPRE.SV40 vector (Addgene, #100054; channelrhodopsin-2 cDNA was replaced). The CAG-floxSTOP-Cx43-P2A-mCherry-Ai6 plasmid was digested with FseI, blunted, and subsequently cut with BsrGI. The resulting 1935 bp fragment was isolated and inserted into the BamHI (blunted) and BsrGI sites of the AAV vector. The resulting pAAV-CAG-Cx43-P2A-mCherry vector was utilized for rAAV2.6-Cx43-P2A-mCherry virus production.

Plasmid DNAs were prepared using the EndoFree Plasmid Maxi Kit (Qiagen). All vectors were verified by sequencing (Eurofins Genomics, Ebersberg, Germany).

### 2.6. Isolation of Murine Neonatal Cardiomyocytes (NNCMs) and Transduction with the AAV2.6-Cx43-P2A-mCherry Virus

Neonatal cardiomyocytes (P1, CD1 background; breeding pairs were supplied by Charles River Laboratories, Sulzfeld, Germany) were isolated (Figure 7b) and enriched using the neonatal heart dissociation and isolation kits (Miltenyi) (for details, see: Raulf, Voeltz et al. [35] and manufacturer’s instructions).

Then, NNCMS were seeded on fibronectin- (0.5 µg/mL, Sigma-Aldrich, Merck, Darmstadt, Germany) coated 96 well glass-bottom plates (Greiner) and cultured in a 20% IMDM medium (30,000 NNCMs/well). After 24 h, FCS was reduced to a 2% IMDM medium (IMDM, 2% *v*/*v* FCS, 100 U/mL/100 µg/mL P/S, 0.1 mM NEAA, and 0.1 mM β-ME), and the virus was added to the medium (0.55 Virus Genomes (VG)/cell). Two days post-transduction, the medium was changed (2% IMDM), and three days post-transduction, mCherry^+^ cells were observed. Transduction efficiency was calculated as a ratio of double positive NNCMs (α-actinin^+^ and mCherry^+^)/all NNCMs (α-actinin^+^) in four fields of view/well (Nikon Eclipse Ti and Nikon A1R MP system, Nikon, Tokyo, Japan) with DAPI, Cy3, and Cy5 filters; 20× objective; and Lasers (LU-N4 laser unit, 405 nm, 488 nm, 561 nm, 640 nm) using NIS-Elements AR 5.11.01 software (Nikon).

### 2.7. Karyotyping of Transgenic G4 ES Cell Clones

Suitable G4 ES cell clones were selected based on karyotyping. Cells were incubated with 0.1 µg/mL demecolcine solution (Sigma-Aldrich, Merck) in a G4 ES cell medium for 3 h, trypsinized (Thermo Fisher Scientific) and treated for 20 min with 75 mM KCl solution (Sigma-Aldrich, Merck); a fixation solution containing methanol (Carl Roth, Karlsruhe, Germany) and acetic acid (Carl Roth) (3:1) was added to preserve chromatin morphology. The suspension was dripped on glass slides (Marienfeld, Lauda-Königshofen, Germany). A Hoechst solution (1 µg/mL in PBS, Sigma-Aldrich, Merck) was used (15 min, RT) for chromosome staining, and microscopic pictures were taken (Axio Observer Microscope, Zen Software 2.6, Carl Zeiss MicroImaging, Inc., Oberkochen, Germany).

### 2.8. Analysis of Transgene Integration into the Rosa26 Locus by Southern Blot

#### 2.8.1. Extraction of Genomic DNA

Total high-molecular-weight DNA of transgenic and WT ES cells was isolated by standard DNA isolation protocol. This consists of lysis in 17.5 mM sodium dodecyl sulfate (SDS), 50 mM Tris (pH 8.0), 10 mM ethylenediaminetetraacetic acid (EDTA), proteinase K (200 µg/mL, Carl Roth); phenol-chloroform-isoamyl alcohol extraction (Sigma-Aldrich, Merck, 25:24:1); chloroform-isoamyl extraction (Sigma-Aldrich, Merck, 24:1); precipitation and resuspension of DNA in 10 mM Tris and 1 mM EDTA, pH 8.0 (TE); RNase treatment (1 mg/mL, Qiagen); further extraction; and precipitation and resuspension of DNA in TE buffer.

#### 2.8.2. Preparation of DNA Probes

The 391 bp Cx43 DNA probe was obtained by a PCR reaction using the CAG-floxSTOP-Cx43-P2A-mCherry-Ai6 construct DNA as a template and the following primer pair: fw: GAGCTCAAAGTGGCGCAGAC and rev: CCTTCACGCGAT CCTTAACG (PCR program: (I) 95 °C for 15 min; 30 cycles of (II-IV): (II) 95 °C for 20 s, (III) 60 °C for 60 s and (IV) 72 °C for 2 min; (V) 72 °C for 10 min; HOT FIREPol Blend Master Mix Ready to Load (Solis Biodyne, Tartu, Estonia)), followed by agarose gel electrophoresis (1% gel) and extraction of the 391 bp DNA band.

For the generation of the Rs26-specific DNA probe (pRs26-5′, see Soriano et al. [36]), the plasmid pDonor MCS Rosa26 vector (Addgene, #37200) was digested with EcoRI, and the resulting 450 bp fragment was isolated. pRs26-5′probe sequence:

CAGGGAAAACGACAAAATCTGGCTCAATTCCAGGCTAGAACCCTACAAATTCAACAGGGATATCGCAAGGATACTGGGGCATACGCCACAGGGAGTCCAAGAATGTGAGGTGGGGGTGGCGAAGGTAATGTCTTTGGTGTGGGAAAAGCAGCAGCCATCTGAGATAGGAACTGGAAAACCAGAGGAGAGGCGTTCAGGAAGATTATGGAGGGGAGGACTGGGCCCCCACGAGCGACCAGAGTTGTCACAAGGCCGCAAGAACAGGGGAGGTGGGGGGCTCAGGGACAGAAAAAAAAGTATGTGTATTTTGAGAGCAGGGTTGGGAGGCCTCTCCTGAAAAGGGTATAAACGTGGAGTAGGCAATACCCAGGCAAAAAGGGGAGACCAGAGTAGGGGGAGGGGAAGAGTCCTGACCCAGGGAAGACATTAAAAAGGTAGTGGGGTCGACTAGATGAAGGAGAGCCTTTCTCTCTGGGCAAGAGCGGTGCAATGGTGTGTAAAGGTAGCTGAGAA

#### 2.8.3. Blotting

For Southern blot analysis, 10 µg genomic DNA were digested with AflIII or double digested with EcoRV and HindIII and the addition of 2.5 mM of spermidine at 37 °C overnight. Enzymes were inactivated by incubation for 20 min at 60 °C and 80 °C, respectively, and digested DNAs were separated overnight (30 V) in 0.8% agarose gels containing 0.01% ethidium bromide. A Lambda HindIII digested DNA marker (Promega, Madison, WI, USA) was used to determine DNA fragment sizes. After 15 min in 0.25 N HCl, 5 min in distilled water, and two 15 min periods in 0.5 M NaOH, the DNA was transferred to a positively charged nylon membrane treated with distilled water and 0.5 M NaOH (BrightStar-Plus, Ambion, Thermo Fisher Scientific) via capillary blotting method (transfer buffer: 1M NaCl and 400 mM NaOH) for 3 h at room temperature. Following this, the membrane was neutralized for 15 min in 2× SSC buffer (0.3 M NaCl and 0.03 M sodium citrate tribasic dihydrate) and dried for 15 min at 80 °C (Heratherm incubator, Thermo Fisher Scientific) to immobilize the DNA. The membrane was then prehybridized for 3 h in a glass hybridization tube (Analytik Jena GmbH, Jena, Germany) placed in a hybridization oven (Analytik Jena GmbH) at 60 °C in an ULTRAhyb ultrasensitive hybridization buffer (Invitrogen, Thermo Fisher Scientific). DNA probes were labelled with α^32^P-dCTP (PerkinElmer 6000 Ci/mmol 20 mCi/mL Lead, 500 µCi, Waltham, MA, USA) using the Prime-It II random primer labeling kit (Agilent Technologies, Santa Clara, CA, USA). Labelled probes were purified with Bio-Spin P30 Tris Chromatography Columns (Bio-Rad) added to the prehybridized membrane, and hybridization was performed under stringent conditions (68 °C) overnight. Filters were washed four times (each 30 min, 55 °C) in 2× SSC containing 0.1% SDS and were exposed to hyperfilm MP8 films (GE Healthcare GmbH, Solingen, Germany) using intensifier screens (Amersham, GE Healthcare). The hyperfilms were exposed for 6–8 days before they were developed and fixed (Kodak GBX Fixer and Replenisher, Sigma-Aldrich, Merck; Kodak GBX Developer and Replenisher, Sigma-Aldrich, Merck; both 1:5 diluted with distilled water).

### 2.9. Selection of Suitable G4 ES and 3T3 Cell Clones Using PCR Analysis

Cells of the different G4 ES and 3T3 clones were lysed, and DNA was extracted using the Gentra Puregene Cell Kit (Qiagen). DNA was amplified applying 5× HOT FIREPol Blend Master Mix Ready to Load (Solis Biodyne) or GoTaqG2 Hot Start Green Master Mix (Promega) and following primer pairs for G4 ES cell clones: (I) integration into the 5′ end of Rs26 locus: fw (endogenous Rs26 sequence): GCCGCCTAAAGAAGAGGCTG, rev (CAG promoter of the insert): GGCGGGCCATTTACCGTAAG; (II) integration into the 3′ end of Rs26 locus: fw (plasmid specific sequence): AGCCTCTGTTCCACATACAC, rev (endogenous Rs26 sequence): GTGCAGTGTTGAGGGCAATC; (III) WT Rs26 allele: fw: AAGGGAGCTGCAGTGGAGTA, rev: CCGAAAATCTGTGGGAAGTC; (IV) detection of Cx43: fw: TGTGGCTGTCGGTGCTCTTC, rev: GAGCCAAGTACAGGA GTGTG; (V) mCherry: fw: AGACCGCCAAGCTGAAGGTG, rev: TCAGCTTCAGCCT CTGCTTGATCT and for 3T3-fibroblasts: (IV) Cx43: fw: TGTGGCTGTCGGTGCTCTTC, rev: GAGCCAAGTACAGGAGTGTG; (VI) Cx43-mCherry: fw: TGTGGCTGTCGG TGCTCTTC, rev: TCAGCTTCAGCCTCTGCTTGATCT.

To test the functionality of the Cre-loxP system RNA of Cre^−^ as well as Cre^+^ G4 ES cell clones 29 and 31 were isolated using the RNeasy Mini Kit (Qiagen), and cDNA was synthesized with the SuperScript VILO cDNA Synthesis Kit (Invitrogen, Thermo Fisher Scientific). CAG-driven expression of the transgene using a forward primer that binds to the first exon of the CAG promoter in combination with a reverse primer binding to Cx43 sequence demonstrated functionality of the Cre-loxP system: fw: CTGACTGACCGCGTTACTC, rev: AGCTGACTCAACCGCTGTCC.

Agarose gel electrophoreses were run to separate PCR products, which were visualized by ethidium bromide (Carl Roth) using an UV light imager Intas GelSTICK Touch (Intas Science Imaging Instruments GmbH, Göttingen, Germany) in combination with Intas GDS Touch 2 software 1.1.5.4 (Intas Science Imaging Instruments GmbH).

### 2.10. Copy Number Determination by qPCR

To analyze actual transgene copy numbers of G4 ES cells, the genomic DNA of three Cre^−^ clones was isolated (EchoLUTION CellCulture DNA kit, BioEchoLife Sciences GmbH, Cologne, Germany) according to manufacturer’s instructions. Then, qPCR was performed as triple determination with 200 ng of genomic DNA, TaqMan Gene Expression Master-Mix (Applied Biosystems, Thermo Fisher Scientific), a Tfrc-VIC housekeeper TaqMan probe (TaqMan Copy Number Reference Assay, mouse, Tfrc, Applied Biosystems, Thermo Fisher Scientific, #4458366), and a custom-made mCherry binding TaqMan probe (Thermo Fisher Scientific). A CFX 96 Real-Time System (Bio-Rad) and 96 well PCR plates (Bio-Rad) with seals (Bio-Rad) (PCR program: (I) 95 °C for 10 min, 35 cycles of (II–IV): (II) 95 °C for 15 s, (III) 60 °C for 30 s, (IV) 72 °C for 30 s) were used. CT values for the target (mCherry) and reference (Tfrc, two copy numbers) gene were computed with Bio-Rad CFX Manager 3.1 software (Bio-Rad). The ∆CTTR values (CT_target_ − CT_reference_) and copy numbers (2^−ΔCTTR^ × 2) of the mCherry-containing transgene were calculated.

### 2.11. RT qPCR

To test for Cx43 (endogenous and endogenous + exogenous), Cx45, Cx40, and Cx30.2 expression, the RNA of G4 ES cells and G4 ES cell-derived EBs (Cre^−^ and Cre^+^) was extracted using the RNeasy Micro Kit (Qiagen), and cDNA was synthesized as described in 2.10. Then, qPCR was performed as triple determination with 25 ng of cDNA, TaqMan probes (Thermo Fisher Scientific) for endogenous Cx43 (exon 1, Mm00439105, Gja1), total (endogenous + exogenous) Cx43 (exon 2 cDNA coding sequence, custom-made), Cx45 (Mm01253027, Gjc1), Cx40 (Mm07294003, Gja5), Cx30.2 (Mm01204089, Gjc3), and the TaqMan Gene Expression Master-Mix (Applied Biosystems, Thermo Fisher Scientific) were used. As internal control GAPDH (Thermo Fisher Scientific, #4352339E) was applied. Then, qPCR was performed on a CFX 96 cycler (Bio-Rad) as described in 2.10 (39 cycles of (II–IV)). CT values for the Cx isotypes and the reference (GAPDH) cDNAs were computed with Bio-Rad CFX Manager 3.1 software (Bio-Rad) to calculate the ∆CTTR values (CT_target_ − CT_reference_).

### 2.12. Immunofluorescence Staining and Microscopy

Cells on coverslips and EBs were fixed for 30 min with 4% FA (PanReac AppliChem). EBs were embedded in Tissue-Tek O.C.T. Compound (Sakura Finetek, Torrance, CA, USA) and cryosectioned (10 µm, Cryostat (Leica, CM 3050S, Wetzlar, Germany)). Prior to staining, cells and EB slices were permeabilized with 0.2% Triton X (Sigma-Aldrich, Merck) in PBS (PBS Tablets, Gibco, Thermo Fisher) for 10 min. For immunostainings, the following antibodies (diluted in 5% donkey serum (Jackson ImmunoResearch, West Grove, PA, USA), 2 h at RT or overnight at 4 °C) were used: Cx43 (1:3000, custom-produced rabbit polyclonal antibody, PSL GmbH, Heidelberg, Germany; immunizing peptide sequence: CDQRPSSRASSRASSRPRPDDLEI [15,37,38]), P2A (1:700, mouse monoclonal IgG1 kappa antibody, Novus Biologicals, Littleton, CO, USA, #NBP2-59627), mCherry (1:5000, chicken polyclonal IgY antibody, Novus Biologicals, #NBP2-25158), α-actinin (1:200, Sigma-Aldrich, Merck, #A7811), and cCasp3 (1:50, Cell Signaling Technology, Danvers, MA, USA, #9661). After several washing steps, secondary antibody incubation was performed (diluted in 1 μg/mL Hoechst/PBS (Sigma-Aldrich, Merck) for 1 h at RT): donkey anti-rabbit IgG (H + L) Alexa Fluor 488/Cy2-conjugated (1:400, AffiniPure, Jackson ImmunoResearch, #711-545-152/#711-225-152); donkey anti-chicken IgY (IgG) (H + L) Cy3 conjugated antibody (1:3000, AffiniPure, Jackson ImmunoResearch, #703-165-155); donkey anti-mouse (IgG) (H+L) Alexa Fluor 647 conjugated antibody (1:3000, AffiniPure, Jackson ImmunoResearch, #715-605-151); and donkey anti-rabbit (IgG) (H + L) Alexa Fluor 647 conjugated antibody (1:3000, AffiniPure, Jackson ImmunoResearch, #711-605-152). After washing, EB slices and cells were mounted with Aqua-Poly/Mount (Polysciences, Warrington, PA, USA). Cells seeded on 96 well glass plates were mounted with PBS. Imaging was performed (I) prior fixation with a fluorescence microscope (Axio Vert A1, Carl Zeiss) using a Cy3 filter, 10× and 20× air objectives, and (II) after fixation and staining, pictures were taken with a confocal microscope (Nikon Eclipse Ti and Nikon A1R MP system, Nikon) with DAPI, Cy2, Cy3, and Cy5 filters, 20×, 40×, and 60× water objectives, Lasers (LU-N4 laser unit (405 nm, 488 nm, 561 nm, 640 nm)) using NIS-Elements AR 5.11.01 software (Nikon).

### 2.13. Western Blot Analysis of Cx43, P2A, and mCherry Protein Expression of Transgenic Cell Lines

Cells were lysed on ice in freshly prepared RIPA buffer (1 mM EDTA (Applichem); 50 mM Tris HCl (pH 7.5) (Carl Roth); 150 mM NaCl (Carl Roth); 0.25% sodium deoxycholate (Sigma-Aldrich, Merck); 1% IGEPAL CA-630 (Sigma-Aldrich, Merck); 1 mM phenylmethanesulfonyl fluoride (Sigma-Aldrich, Merck); Protease and Phosphatase Inhibitor (Thermo Fisher)). SDS mini gels were cast (10% separating gel: 10% acrylamide (Bio-Rad) in 375 mM Tris HCl (pH 8.8), 0.1% SDS (Carl Roth), 0.05% APS (Bio-Rad), 0.05% TEMED (Sigma-Aldrich, Merck); 5% stacking gel: 5% acrylamide in 125 mM Tris HCl (pH 6.8), 0.1% SDS, 0.05% APS, 0.1% TEMED). Protein lysates were mixed 1:1 with 2× Laemmli Buffer (Bio-Rad) supplemented with β-ME (Sigma-Aldrich, Merck) and denatured for 10 min at 100 °C. Protein samples and Precision Plus Protein WesternC Standards (Bio-Rad) separated by gel electrophoresis (ProSieve EX Running Buffer, Lonza, Basel, Switzerland) were transferred onto a methanol-activated (Carl Roth) 0.22 µm PVDF-FL membrane (Biozym) by tankblot (ProSieve EX Western Blot Transfer Buffer, Lonza). Non-specific binding sites of membranes were blocked for 1 h with 5% skim-milk powder (VWR) solved in TBST (20 mM Tris (Sigma-Aldrich, Merck), 150 mM NaCl, 0.1% Tween-20 (Applichem)). Membranes were incubated with Cx43 (1:3000, custom-produced rabbit polyclonal antibody, PSL GmbH, Heidelberg, Germany; 1:8000, rabbit polyclonal antibody, Sigma-Aldrich, Merck, #C6219), P2A (1:700, mouse monoclonal IgG1 kappa antibody, Novus Biologicals, #NBP2-59627), or mCherry (1:5000, chicken polyclonal IgY antibody, Novus Biologicals, #NBP2-25158) antibodies over night at 4 °C. After washing with TBST (3 × 5 min, RT), they were incubated for 1 h at RT with a donkey anti-rabbit IgG (H+L) Alexa Fluor 488 conjugated antibody (1:3000, AffiniPure, Jackson ImmunoResearch, #711-545-152), a goat anti-mouse IgG peroxidase conjugated antibody (1:3000, Sigma-Aldrich, Merck, #A2304), a donkey anti-chicken IgG (H+L) Alexa Fluor 647 conjugated antibody (1:3000, AffiniPure, Jackson ImmunoResearch, #703-175-155), and an Alexa 555 conjugated-GAPDH loading control monoclonal antibody (1:5000, Thermo Fisher, #MA5-15738). Blots with peroxidase-conjugated secondary antibodies were incubated with Pierce ECL Western Blotting Substrate (Thermo Fisher). First and secondary antibodies were diluted in 5% skim milk powder solved in TBST. All blots were detected with the ChemiDoc MP Imaging System (Bio-Rad). Image Lab 5.2.1 software (Bio-Rad) was used for semi-quantitative analysis of Cx43 protein expression, which was normalized to GAPDH as housekeeper.

### 2.14. Dye Transfer Experiments in HeLa Cells

Cells were seeded on 0.1% gelatin- (Merck) coated cover glasses (VWR) placed in 24-well plates containing 20% DMEM medium plus additives, as described above. A small blue fluorescent Alexa Fluor 350 Hydrazide dye (349 Da, Invitrogen, Thermo Fisher Scientific, #A10439) and a large red fluorescent dextran-coupled Alexa Fluor 647 dye (10 kDa, Invitrogen, Thermo Fisher Scientific, #D22914) were diluted to a concentration of 0.5 µg/µL in intracellular solution (40 mM KCl (Sigma-Aldrich, Merck), 90 mM KAsp (Sigma-Aldrich, Merck), 10 mM NaCl (Carl Roth), 3 mM Mg-ATP (Sigma-Aldrich, Merck), 1 mM MgCl (Sigma-Aldrich, Merck) and 10 mM HEPES (Sigma-Aldrich, Merck) and adjusted to pH 7.4 with KOH (Sigma-Aldrich, Merck)). HeLa cells were dialyzed with a patch clamp pipette (BF 150-86-10, Science Products, Hofheim, Germany) and filled with the above-mentioned solutions under microscopic control. When the patch pipette touched the cell, a bright field and a fluorescence picture were taken (a DAPI filter (F36 500 HC, Nikon) for Alexa Fluor 350 or a Cy5 filter (F 46-009 ET, Nikon) for Alexa Fluor 647 (20× Objective Fluor, Nikon)). The membrane resistance was monitored through an EPC10 amplifier (HEKA Elektronik GmbH, Reutlingen, Germany) until reaching whole cell configuration in the voltage clamp mode (holding potential: −40 mV). Fluorescence pictures were taken after obtaining whole-cell configuration at 0.5 min, 5 min, and 10 min. Pictures were taken with the Grasshopper 3 Camera (GS3-U3-23S6C-C, Teledyne Flir LCC, Wilsonville, OR, USA) controlled by the Micro-Manager 2.0 beta software (Image J, Bethesda, CA, USA).

### 2.15. Fluorescence Recovery after Photobleaching (FRAP)

3T3-fibroblasts and G4 ES cells were incubated for 20 min at 37 °C with calcein AM (0.38 µM, Invitrogen, Thermo Fisher Scientific, #C3100MP) diluted in 10% DMEM medium (3T3, see above) or 15% KnockOut-DMEM (G4 ES cells, see above). Fluorescence intensity of a cluster of calcein^+^ 3T3-fibroblasts or G4 ES cells was measured using a confocal microscope (Nikon Eclipse Ti, 40× objective; CFI Apo Lambda S LWD 40XWI, NA 1.25, Nikon). Next, a single fibroblast or G4 ES cell in the cluster was bleached with a 561 nm laser pulse (5 s, 2.5 mW), fluorescence intensity was measured, and pictures were taken over 10 min at 15 s intervals. Fluorescence intensities were converted into values between 1 (fluorescence intensity before bleaching) and 0 (fluorescence intensity immediately after bleaching).

### 2.16. Video Microscopy of Cell Clusters of Dissociated Beating EBs

Cre^+^ and Cre^−^ beating EBs were harvested at d12/d13 of differentiation. After partial dissociation with trypsin for 20 min at 37 °C, cell clusters were seeded on 0.1% gelatin- (Merck) coated 24-well plates filled with 20% IMDM medium. One day later, frequency measurements were performed via video microscopy of beating clusters on a Nikon Eclipse Ti2 inverted microscope system with a 4× objective (Fluor, Nikon) using a brightfield setting. EB clusters plated in the 24 wells were heated by an Ibidi temperature controller (Ibidi GmbH, Gräfelfing, Germany), and CO_2_ was maintained at 5% through an Ibidi gas mixer (Ibidi GmbH). Spontaneous contractions were recorded with a Grasshopper 3 Camera (GS3-U3-23S6C-C, Teledyne Flir LCC) analyzed online and offline using a custom-designed software (LabView, National Instruments, Austin, TX, USA), as described earlier in Makowka, Bruegmann et al. [39]. Time points of individual beats were recorded by a PowerLab system through the sound recorder of the PC (PowerLab 4/35 and Labchart 8 software, AD Instruments, Sydney, Australia) and used to calculate the frequency in offline analysis. For statistical analysis, the mean of beats per min (bpm) and its standard deviation (SD) of 5 min recording (sampling every second) was calculated. The mCherry expression of Cre^+^ cell clusters was verified before the experiment with a Cy3 filter (F 46-004 ET, Nikon) using a prime BSI camera (Teledyne Photometrics, Tuscon, AZ, USA) controlled by the NIS-Elements AR 5.21.03 software (Nikon).

### 2.17. Statistical Analyses

Relative Cx43 expression data of Western blots were either compared using an unpaired two-tailed *t*-test (Cre^+^ vs. Cre^−^ ES cells) or an ordinary one-way ANOVA test followed, in case of significance, by a Tukey’s multiple comparisons test (comparison of WT-, Cre^−^-, and Cre^+^ 3T3-fibroblasts). Ordinary one-way ANOVA and Tukey’s multiple comparisons tests (comparison of WT-, Cre^−^-, and Cre^+^ 3T3-fibroblasts) or an unpaired two-tailed *t*-test (Cre^+^ vs. Cre^−^ ES cells) were determined to evaluate statistical differences of FRAP experiments. Beating frequencies and standard deviations of beating frequencies were compared using an unpaired two-tailed *t*-test. Cx isoform expression of RT qPCR analysis was compared using an unpaired two-tailed *t*-test (Cre^+^ vs. Cre^−^ EBs). All error bars are provided as SEMs. Statistical significance was considered a *p*-value of ≤0.05. All statistical analyses were calculated using GraphPad Prism 9.3.0 (GraphPad Software Inc., San Diego, CA, USA).

## 3. Results

### 3.1. Generation and Characterization of Inducible Cx43 Overexpression in Mouse G4 ES Cell Lines

**Figure 1:**
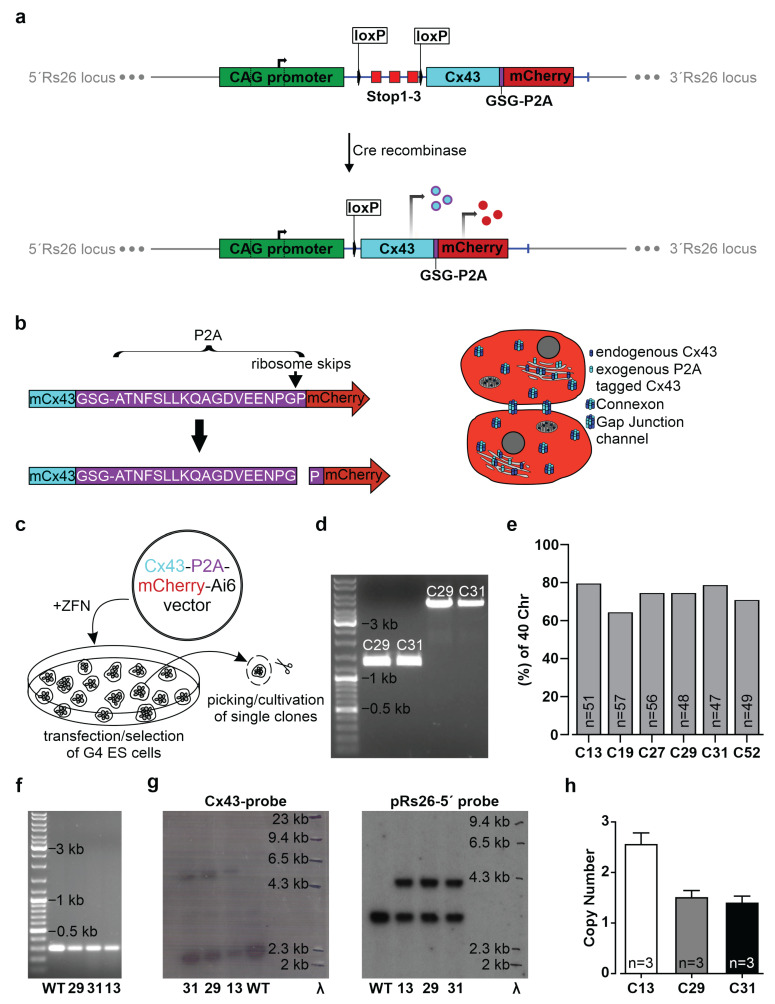
Generation and characterization of transgenic mouse G4 ES cells with inducible overexpression of connexin (Cx) 43. (**a**) Scheme of the homologous integrated CAG-floxed-Cx43-P2A-mCherry-fragment into the mouse Rs26 locus, enabling the inducible overexpression of Cx43-P2A and the fluorescent protein mCherry. Upon Cre recombinase activity, the loxP flanked stop sites are excised and exogenous Cx43, which is C-terminal-tagged with 21 amino acids of P2A, and mCherry are expressed under control of the CAG-promoter. (**b**) Principle of Cx43 and mCherry co-expression by using the self-cleaving peptide P2A (left side). Transgene expression and Cx43 gap junction formation are depicted schematically in two adjacent cells (right side). (**c**) Scheme of the co-transfection of murine G4 ES cells with the expression vector and zinc finger nucleases and the selection approach of suitable clones. (**d**) PCR analysis of the Rs26 locus revealed correct integration of the transgene. The expected 1325 bp and 4636 bp fragments for the 5′Rs26- and the 3′Rs26 integration, respectively, could be detected. (**e**) Karyotyping of G4 ES cell clones (C) 13, 19, 27, 29, 31, and 52 yielded varying percentages of cells with 40 chromosomes (Chr). (**f**) PCR analysis showed at least one WT allele of the Rs26 locus, indicating a single integration of the transgene. (**g**) Southern blot analysis with α^32^PdCTP labeled probes confirmed the integration of the exogenous Cx43 expression cassette (exogenous Cx43: 5583 bp; endogenous Cx43: 2304 bp, left picture) into the Rs26 locus (integration of the transgene into Rs26: 4171 bp; WT Rs26: 3050 bp, right picture). (**h**) qPCR-based determination of the copy number of the transgene in individual clones: G4 ES cell clones 29 and 31 were found to carry a single copy, whereas clone 13 most likely carries a second copy outside of the Rs26 locus.

We have opted to generate an inducible Cx43 overexpression system in pluripotent mouse embryonic stem (ES) cells using a fluorescent reporter (mCherry), marking the overexpressing cells and a small detectable peptide (P2A), which is fused to the C-terminus of exogenous Cx43 (Figure 1b). For this purpose, we have generated mouse G4 ES cells, in which the CAG promoter drives the inducible overexpression of Cx43. The CAG promoter was chosen because of its strong expression in muscle cells [34,40,41] at all stages of mouse embryonic development and after birth. The inducibility of the expression cassette is provided by a loxP flanked stop cassette with three SV40 polyadenylation signals that can be removed by Cre protein activity (Figure 1a). As depicted in Figure 1a, a DNA fragment composed of the murine Cx43 cDNA followed by the GSG-P2A DNA-sequence and the mCherry cDNA in frame was cloned downstream of the stop cassette of the Ai6 vector (CAG-floxSTOP-Cx43-P2A-mCherry-Ai6) (Figure 1a,b). This vector contains Rosa26 (Rs26) genomic sequences to drive homologous recombination of the expression cassette into the endogenous Rs26 locus of G4 ES cells, allowing targeted single integration (Supplementary Figure S1a) [42].

To obtain a single integration into the genomic Rs26 locus, G4 ES cells were co-transfected with this construct and specific zinc finger nucleases (Figure 1c and Supplementary Figure S1a). We have characterized the transfected cells and assessed successful single integration of the transgene into the Rs26 locus of individual cells with various approaches: First, ten (52 clones in total) independent transgenic G4 ES cell clones were isolated after ten days of selection with neomycin and further cultivated (Figure 1c). The mCherry DNA could be detected in nine out of ten analyzed ES cell clones (Supplementary Figure S1b). PCR analysis of the Rs26 locus integration site using primer pairs that bind to the endogenous Rs26 sequence at the 5′- or 3′-end and to sequences within the construct (Supplementary Figure S1e) demonstrated the correct integration of the construct into the Rs26 locus of clones 13, 19, 27, 29, 31, 52 (Supplementary Figure S1c; as representative examples, clones 29 and 31 are shown in Figure 1d). Karyotyping of these six different cell clones revealed best results for clones 13, 29, and 31, as the large majority of counted cells (80% of clone 13, 75% of clone 29 and 79% of clone 31) contained 40 chromosomes (Figure 1e).

In order to investigate whether one or both Rs26 alleles were affected by integration of the insert, a Rs26-specific primer pair was used. PCR analysis resulted in a 297 bp band, indicating one unaffected Rs26 allele (Figure 1f). We also performed Southern blot analysis, proving the integration of the entire expression cassette (Figure 1g, left picture; Supplementary Figure S1f) into one allele of the Rs26 locus (Figure 1g, right picture; Supplementary Figure S1f) and confirming one WT allele for each tested clone and one integration of the transgene. Then, we used qPCR to confirm single integration of the transgene in individual clones (Figure 1h). These experiments revealed that G4 ES cell clones 29 and 31 carry one copy of the transgene. We also found that clone 13 most likely carries a second copy (Figure 1h) that appeared integrated outside of the Rs26 locus, as one remaining WT Rs26 allele could be detected by Southern blot analysis (using the pRs26-5′ probe) (Figure 1g; Supplementary Figure S1f), as well as by qualitative PCR (Figure 1f; Supplementary Figure S1e). We therefore used for the further experiments clones 29 and 31.

**Figure 2:**
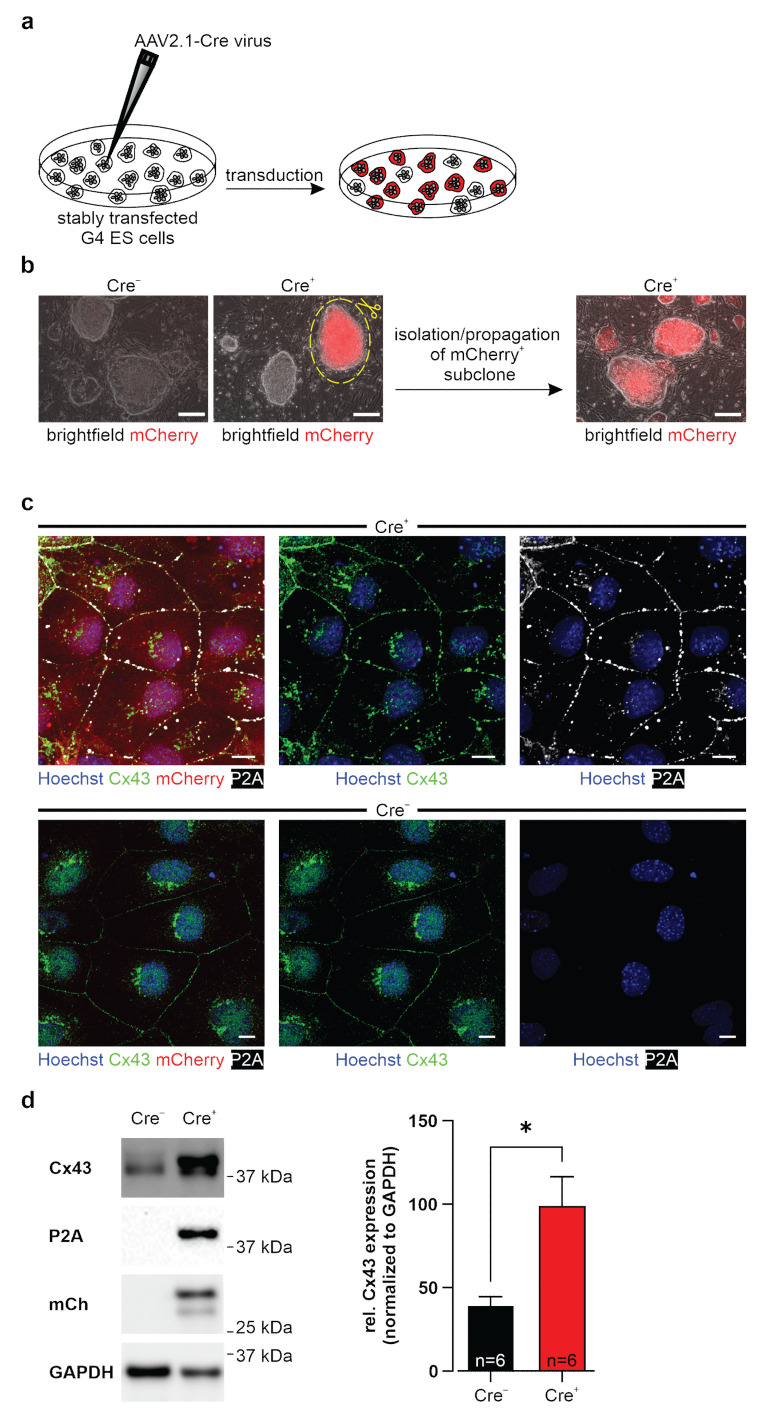
Inducible Cx43 overexpression in stably transfected, undifferentiated murine G4 ES cells. (**a**) Stably transfected (CAG-floxSTOP-Cx43-P2A-mCherry-Ai6) G4 ES cells of clones 29 and 31 (Cre^−^) were transduced with an AAV2.1-Cre virus (Cre^+^) to induce transgene expression. (**b**) Transgenic G4 cells prior to AAV2.1-Cre transduction were mCherry^−^ (left picture, shown for C31), and three days after transduction, most of the colonies were mCherry^+^ (middle picture). Single subclones with strong mCherry expression were isolated, and cells were further cultivated (middle and right pictures, shown for C31). Scale bars 100 µm. (**c**) Immunostainings of Cre^+^ G4 ES cells (subclone 31, upper panel) showed, in contrast to Cre^−^ control cells (C31, lower panel), prominent expression of mCherry and of membrane-associated exogenous Cx43 (P2A^+^). Total Cx43 (green), P2A-tagged exogenous Cx43 (white), mCherry (red), and Hoechst (blue). Scale bars 10 µm. (**d**) Western blot analysis of Cre^−^ and Cre^+^ cells of C31 proved inducible Cx43 overexpression; note also mCherry^−^ and P2A expression in the AAV2.1-Cre treated cells. * *p* value ≤ 0.05.

Next, we assessed the functionality of the Cre-loxP system by transducing the undifferentiated murine G4 ES cells of clones 29 and 31 with AAV2.1-Cre (Figure 2a). Cell suspensions of both transgenic clones were mixed with AAV2.1-Cre and plated on irradiated fibroblasts. After three days several, mCherry-expressing colonies were visible, demonstrating that the floxed stop cassette was excised by the Cre recombinase activity. Since not all cells were successfully transduced, we obtained a mixed cell population containing mCherry positive (Cre^+^) and mCherry negative (Cre^−^) colonies (Figure 2b, middle picture). To obtain homogenous Cre^+^ transgenic cell lines, we isolated single Cre^+^ subclones and expanded these in culture (Figure 2b, middle and right pictures). All these cells were found to be mCherry^+^, whereas the control cells (Cre^−^), not AAV2.1-Cre transduced, were mCherry^−^ (Figure 2b). The functionality of the Cre-loxP system was further corroborated by RT-PCR. Using a primer pair matching exon 1 of the chicken beta actin (CAG promoter) and the Cx43 cDNA, we could show that the stop cassette was removed and the chimeric intron was spliced out (band of 417 bp; Supplementary Figure S1d,e). Thus, both transgenic G4 ES clones C29 and C31 were functioning; for the following experiments, we used the (Cre^−^) and (Cre^+^) cells of clone 31.

We explored the Cx43, mCherry, and P2A expression patterns in ES cells using immunostainings (Figure 2c) and Western blot analysis (Figure 2d and Supplementary Figure S7a). These experiments evidenced that P2A-tagged Cx43 and mCherry could be detected in the Cre^+^ but not in the Cre^−^ control ES cell lines (Figure 2c,d). Based on staining for P2A, we found that the cellular distribution pattern of the exogenous Cx43 protein was almost identical to the endogenous one: for both components, cytoplasmic and membrane location were observed (Figure 2c). Importantly, quantification of Cx43 expression in Western blot analysis proved an approximately 2.5-fold overexpression following Cre-induction upon normalization with GAPDH (Figure 2d; n = 6). In contrast, immunostainings and Western blot analysis in Cre^−^ control cells showed lack of P2A and mCherry expression, underscoring appropriate function of the loxP stop cassette without major leakiness (Figure 2c,d). Taken together, we could generate an inducible Cx43 overexpressing G4 ES cell line carrying a single copy of the CAG-floxSTOP-Cx43-P2A-mCherry construct, which is integrated into the Rosa26 locus.

### 3.2. Formation of Functional Cx43 Gap Junction Channels after Expression of the Inducible Construct in HeLa Cells, 3T3-Fibroblasts, and Undifferentiated G4 ES Cells

**Figure 3:**
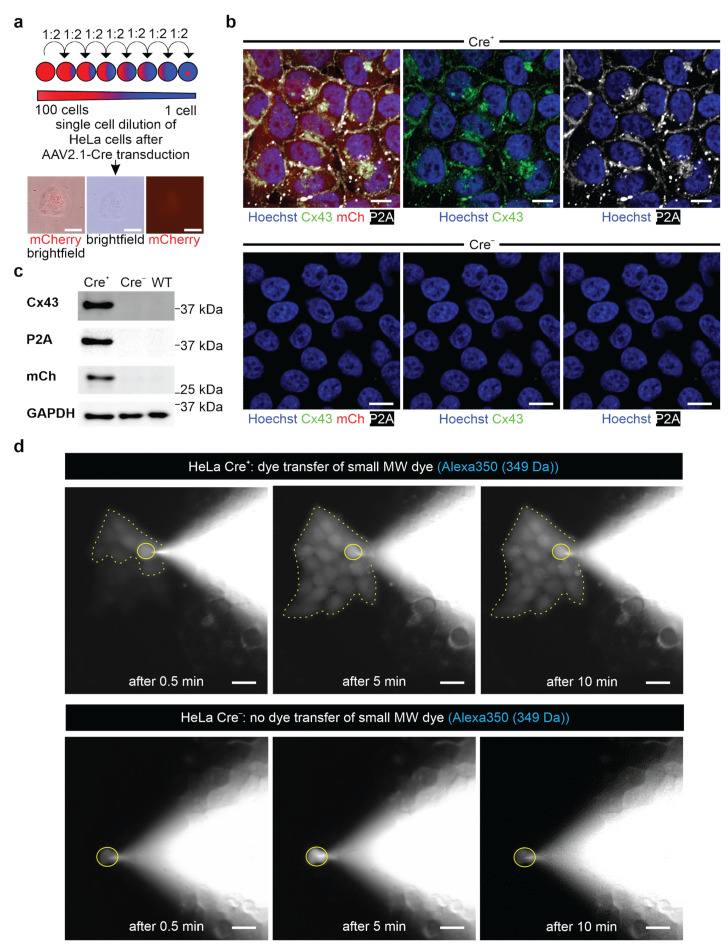
Inducible Cx43 expression and functional gap junction formation in stably transfected HeLa cells. (**a**) Scheme of single cell dilution of stably transfected (CAG-floxSTOP-Cx43-P2A-mCherry-Ai6, Cre^−^) and transduced (AAV2.1-Cre, Cre^+^) HeLa cells. Scale bars 100 µm. (**b**) Immunostainings of transgenic HeLa cells confirmed strong expression of exogenous Cx43 and P2A in the plasma membrane of mCherry^+^ cells (Cre^+^, upper panel), whereas not-transduced control cells were Cx43^−^, P2A^−^, and mCherry^−^ (Cre^−^, lower panel). Scale bars 10 µm. (**c**) Western blot analysis confirmed expression of Cx43, P2A, and mCherry in Cre^+^ HeLa cells, whereas none of these proteins were detected in Cre^−^ and WT HeLa cells. (**d**) Single Cre^+^ or Cre^−^ HeLa cells were dialyzed via a patch pipette with the small MW dye Alexa350 (349 Da), and dye passage to closely adjacent cells was observed in Cre^+^ cells (upper panel; n = 4). Cre^−^ HeLa control cells did not show passage of Alexa 350 to adjacent cells (lower panel, n = 5). Dialyzed cell marked by a yellow circle; after dye diffusion positive cell colonies marked by a yellow dotted line. Scale bars 100 µm.

Even though we could demonstrate inducible overexpression of Cx43 protein in pluripotent cells, this was no proof for functional gap junction formation. This is a critical issue, as tagged gap junction channels were reported to show altered cell biological properties [43,44]. Therefore, we established a transgenic HeLa cell line using the construct described above to assess gap junction functionality. We chose HeLa cells because they do not express endogenous gap junction channels. WT HeLa cells were transfected with the CAG-floxSTOP-Cx43-P2A-mCherry-Ai6 construct and subsequently selected with neomycin for twelve days. Single HeLa cell clones were picked, and transgenic HeLa cell lines (Cre^−^) established by propagating the cells. Next, cells of a transgenic HeLa cell line (Cre^−^) were transduced with the AAV2.1-Cre virus, and mCherry^+^ HeLa colonies could be observed after three days. To establish Cx43 expressing Cre^+^ HeLa cell lines, we performed single cell dilution (Figure 3a). The lines were further characterized by immunostainings and Western blot analysis for Cx43, P2A, and mCherry (Figure 3b,c). We found that Cre^+^ HeLa cells co-expressed P2A-tagged Cx43 protein, which was localized in the cytoplasm and the cell membrane. We also observed that the Cre^−^ transgenic control cells neither displayed mCherry fluorescence nor Cx43 or P2A expression (Figure 3b). These findings were underscored by the Western blot analysis (Figure 3c).

Given that we could generate inducible Cx43 expression in HeLa cells, we next explored functional Cx43 gap junction formation using fluorescent dye diffusion. To discriminate between gap junctions and cytosolic bridges, we used two different dyes: Alexa 350 with a MW of 349 Da and Alexa 647 dextran with a MW of 10 kDa, both at a concentration of 0.5 µg/µL. Single cells were dialyzed with the respective dye using a patch clamp pipette under microscopic control, and we found that Alexa 350 had diffused within 0.5 min to several Cre^+^ HeLa cells, that were in close contact. Within approximately 5 min, the whole cell colony (10–20 cells) was found to be loaded with fluorescent dye (Figure 3d, upper panel, time-dependent dye transfer, n = 4). In contrast, in Cre^−^ HeLa cells, no small MW dye diffusion to adjacent cells could be observed (n = 5) (Figure 3d, lower panel). In addition, the large fluorescent Alexa 647 dextran dye diffused to neither the neighboring Cre^+^ (n = 4) (Supplementary Figure S2a, upper panel) nor the Cre^−^ HeLa cells (n = 3) (Supplementary Figure S2a, lower panel). These experiments proved inducible formation of functional Cx43 gap junction channels.

**Figure 4:**
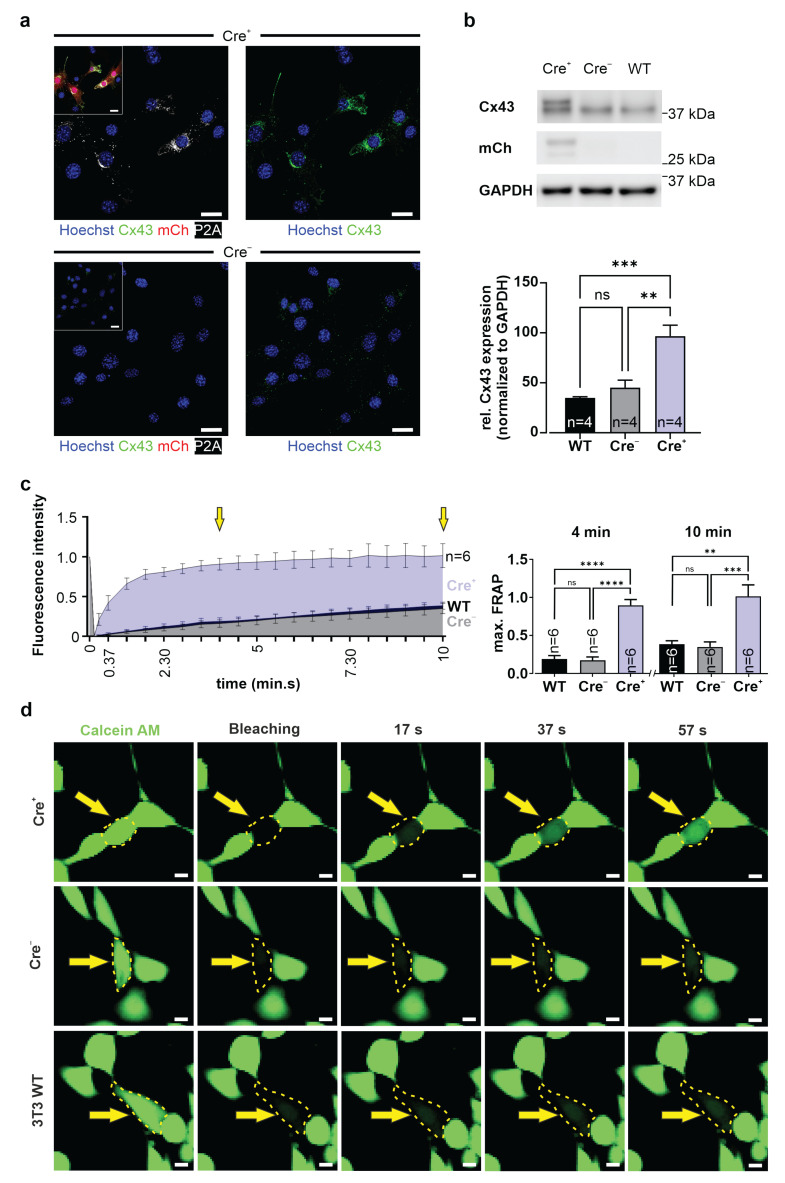
Inducible overexpression of functional Cx43 gap junctions in stably transfected 3T3 cells. (**a**) Immunostaining of stably transfected (CAG-floxSTOP-Cx43-P2A-mCherry-Ai6, Cre^−^) and transduced (AAV2.1-Cre, Cre^+^) mCherry^+^ 3T3-fibroblasts showed strong Cx43 and P2A expression (upper panel). In contrast, Cre^−^ 3T3 cells exhibited much lower Cx43 expression and expressed neither mCherry fluorescence nor P2A. Upper and lower left pictures: Hoechst (blue) and P2A (white); insets: Hoechst (blue), Cx43 (green), mCherry (red), and P2A (white). Scale bars 20 µm. (**b**) Western blot analysis of transgenic Cre^+^ cells proved Cx43 and mCherry (over-)expression but not in control cells. Quantification of Cx43 expression proved its strong overexpression in Cre^+^ 3T3 cells. (**c**) Fluorescence recovery after photobleaching (FRAP) measurements illustrated significantly faster calcein AM dye recovery in transgenic Cre^+^ 3T3-fibroblasts (clone 2) than in either WT or Cre^−^ 3T3-fibroblasts (clone 2); FRAP recovery rates were quantified at 4 and 10 min after bleaching (quantification times are marked by yellow arrows). (**d**) Original fluorescence pictures of 3T3 Cre^+^, Cre^−^, and WT fibroblasts, respectively, after calcein AM (0.38 µM) dye loading; bleaching (for 5 s with a 561 nm laser with 2.5 mW intensity) of a single fibroblast (bleached cell marked by a yellow dotted line and a yellow arrow); and dye recovery at 17 s, 37 s, and 57 s after bleaching; calcein AM dye (green). Scale bars 20 µm. ** *p* value ≤ 0.01, *** *p* value ≤ 0.001, **** *p* value ≤ 0.0001, ns (not significant) *p* value > 0.05.

Besides HeLa cells, we also tested transfection and transduction of 3T3 mouse fibroblasts, which are known to endogenously express a low density of Cx43 gap junction channels. These experiments were performed to investigate inducible Cx43 gap junction channel formation in the presence of endogenous Cx43 gap junctions. 3T3 cells were stably transfected with the CAG-floxSTOP-Cx43-P2A-mCherry-Ai6 vector and selected with neomycin for twelve days. Through single cell dilution, a new transgenic 3T3 line was established (Cre^−^), as proven by PCR analysis (Supplementary Figure S3a). Cells were transduced with AAV2.1-Cre, and three days after transduction, mCherry^+^ 3T3 cells could be detected (Supplementary Figure S3b). Immunostainings of the Cre^+^ and Cre^−^ 3T3-fibroblasts proved, as would be expected, large amounts of Cx43 and P2A (Figure 4a, upper panel) in the mCherry^+^ cells but no P2A and only low amounts of endogenous Cx43 in the controls (Figure 4a, lower panel). The mCherry and Cx43 overexpression in Cre^+^ 3T3-fibroblasts compared to control cells was confirmed by Western blot analysis (n = 4) (Figure 4b). We also studied functional Cx43 gap junction formation using fluorescence recovery after photobleaching (FRAP), which enables, in contrast to dye diffusion, quantification of the diffusion velocity of the calcein AM dye. Cells were first loaded with calcein AM, followed by the bleaching of a single fibroblast in a cell cluster using a 561 nm laser for 5 s with an intensity of 2.5 mW. Thereafter, recovery of the fluorescence intensity in the bleached fibroblast was determined every 15 s over a period of 10 min (Figure 4c,d). Our experiments showed that Cre^+^ 3T3-fibroblasts displayed significantly faster fluorescence recovery after photobleaching compared to Cre^−^ cells (Tukey’s multiple comparisons test after 4 min: *p*(Cre^−^ vs. Cre^+^) < 0.0001; after 10 min: *p*(Cre^−^ vs. Cre^+^) = 0.0007; n = 6) and 3T3 WT cells (Tukey’s multiple comparisons test after 4 min: *p*(WT vs. Cre^+^) < 0.0001; after 10 min: *p*(WT vs. Cre^+^) = 0.001; n = 6), respectively. In contrast, WT and Cre^−^ cells displayed very similar FRAP characteristics (Tukey’s multiple comparisons test after 4 min: *p*(WT vs. Cre^−^) = 0.9523, ns; after 10 min: *p*(WT vs. Cre^−^) = 0.9651, ns; n = 6) (Figure 4c,d; Supplementary Videos S1, S3, and S4). We also noticed that mCherry fluorescence was bleached and could not be recovered, as this protein is too big (28 kDa) to diffuse via gap junctions (Supplementary Figure S3c; n = 6, Supplementary Video S2). These experiments proved an increase in functional gap junction channels by overexpression of exogenous Cx43 in the presence of endogenous Cx43 gap junctions.

**Figure 5:**
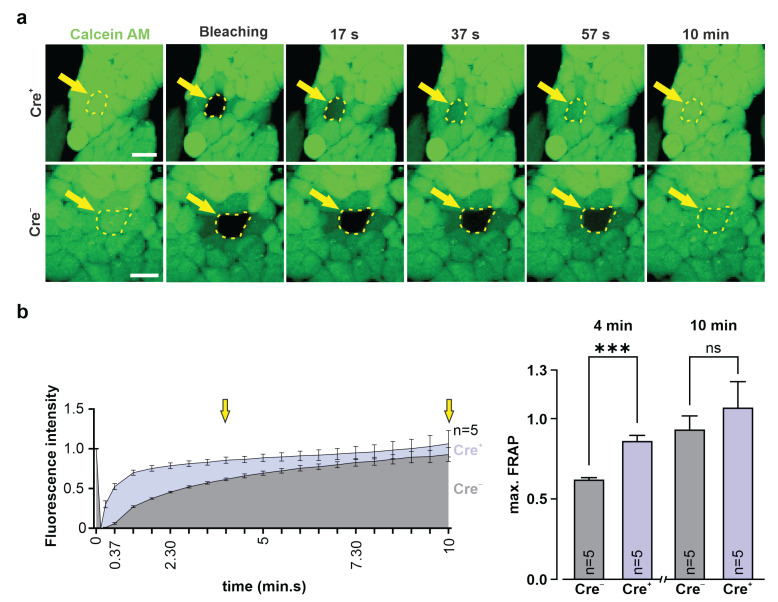
Functional gap junction formation in Cre^+^ G4 ES cells. Fluorescence recovery after photobleaching (FRAP) experiments in G4 ES cells. (**a**) Original fluorescence pictures of Cre^+^ and Cre^−^ G4 ES cells after calcein AM (0.38 µM) dye loading; bleaching (for 5 s with a 561 nm laser with 2.5 mW intensity) of a single G4 ES cell of the cell cluster (bleached cell marked by a yellow dotted line and a yellow arrow); and dye recovery at 17 s, 37 s, 57 s, and 10 min after bleaching; calcein AM dye (green). Scale bars 20 µm. (**b**) FRAP experiments of G4 ES cells showed, 4 min after bleaching, significantly faster calcein AM dye recovery in transgenic Cre^+^ G4 ES cells (clone 31) than in Cre^−^ G4 ES cells (clone 31); after 10 min, there was no significant difference in the dye recovery of both cell lines detected (quantification times are marked by yellow arrows). *** *p* value ≤ 0.001, ns (not significant) *p* value > 0.05.

We also tested the functional exogenous Cx43 gap junction formation in undifferentiated Cre^+^ G4 ES cells using FRAP and compared the results with Cre^−^ control ES cells. Our experiments revealed, at 4 min after bleaching, a significantly faster dye recovery in the Cre^+^ G4 ES cells than in the controls (two-tailed unpaired *t*-test after 4 min: *p*(Cre^+^ vs. Cre^−^) = 0.0005; n = 5), whereas at later time points, the controls also showed a clear fluorescence recovery (two-tailed unpaired *t*-test after 10 min: *p*(Cre^+^ vs. Cre^−^) = 0.4905; n = 5) (Figure 5, Supplementary Videos S5 and S7) due to prominent endogenous Cx43 expression (Figure 2c, lower pictures). Owing to its large size, mCherry fluorescence could not be recovered, as already mentioned above (Supplementary Figure S4a, Supplementary Video S6).

### 3.3. In Vitro Differentiation of Cx43-overexpressing ES Cells into Spontaneously Beating Cardiomyocytes

**Figure 6:**
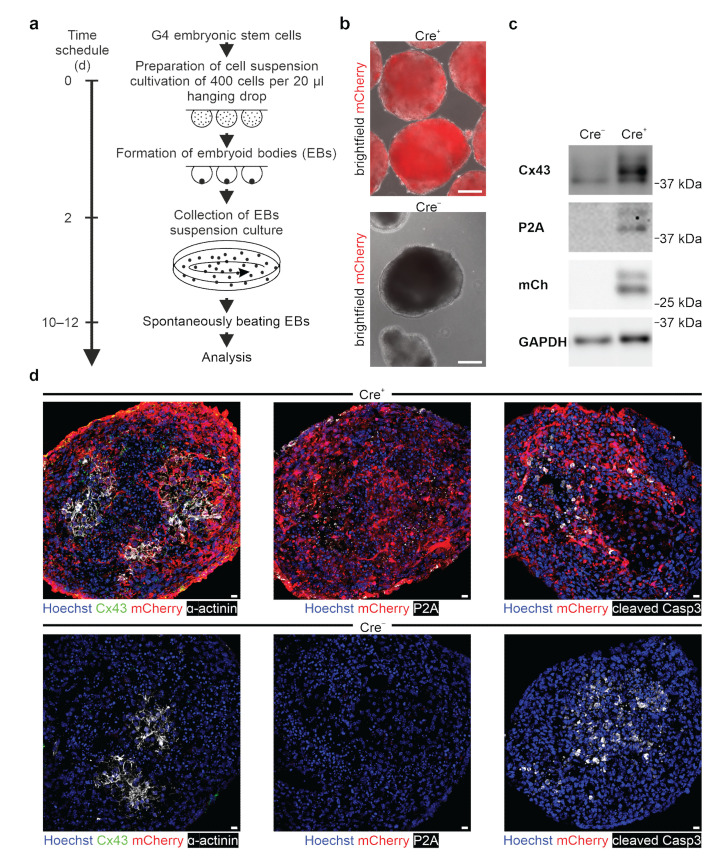
Inducible Cx43 (over-)expression in stably transfected murine G4 ES cells (clone 31), in vitro differentiation into cardiomyocytes. (**a**) In vitro differentiation scheme of murine ES cells into spontaneously beating EBs (adapted from Boheler et al. [33]). (**b**) The mCherry^+^ EBs derived from the transgenic Cre^+^ G4 ES cells of clone 31 (upper picture) and transgenic mCherry^−^ control EBs (lower picture). Scale bars 100 µm. (**c**) Western blot analysis of EBs from Cre^+^ and Cre^−^ G4 ES cells confirmed inducible Cx43 overexpression, as underscored by P2A-tagged Cx43 and mCherry expression. (**d**) Immunostainings of EBs illustrated clusters of cardiac α-actinin^+^ (white) cardiomyocytes in both Cre^+^ (upper left picture) and Cre^−^ control EBs (lower left picture). Immunostainings yielded strong Cx43 (green, upper left picture), P2A (white), and mCherry (red) (over-)expression (upper middle picture) in Cre^+^ EBs. Similar apoptotic areas preferentially in the center of Cre^+^ and Cre^−^ control EBs (right pictures) were found. Scale bars 10 µm.

**Figure 7:**
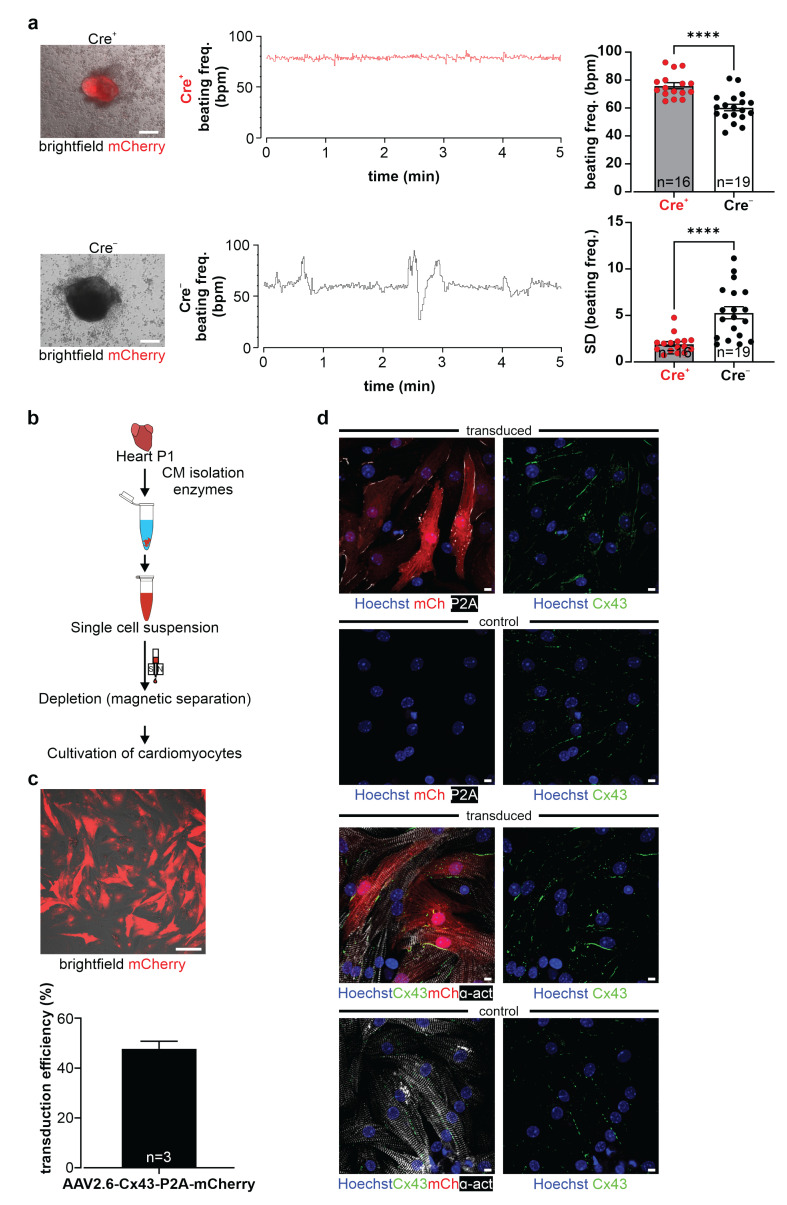
Functional impact of Cx43 overexpression in murine G4 ES cell-derived EBs; targeting of murine neonatal cardiomyocytes (NNCMs) with an AAV2.6-Cx43 overexpression virus. (**a**) Fluorescent and brightfield pictures of Cre^+^ and Cre^−^ EB-derived cell clusters at one day after dissociation and plating. Scale bars 100 µm. Video-based monitoring of spontaneous beating rates showed that Cre^+^ cell clusters (red) exhibited significantly higher and more stable beating rates compared to Cre^−^ controls (black). **** *p* value ≤ 0.0001. (**b**) Scheme depicting the isolation and enrichment of NNCMs. (**c**) NNCMs were cultivated and transduced with the AAV2.6-CAG-Cx43-P2A-mCherry (AAV2.6-Cx43) virus; note strong mCherry fluorescence (picture). Transduction efficiency of AAV2.6-Cx43 virus in NCCMs (graph). (**d**) Immunostainings of transduced NNCMs showed Cx43 (green), P2A (white), and mCherry (red) expression (upper panel), whereas non-transduced NNCMs displayed less Cx43 and neither P2A nor mCherry (second panel) expression. The mCherry^+^(red) and cardiac α-actinin^+^ (white cross-striation) immunostainings (third panel) proved successful targeting of NNCMs with the AAV2.6-Cx43 virus; non-transduced NNCMs showed α-actinin, but less Cx43 staining and no mCherry signal. NNCMs lack intercalated discs; note the diffuse expression pattern of Cx43 in the cell membrane. Scale bars 10 µm.

A key advantage of using pluripotent cells as a Cx43 overexpression system is that they can be differentiated in vitro, and the consequences of overexpression can be tested regarding development, differentiation, and function in different somatic cell types. As a proof of concept, we have focused on the in vitro differentiation into cardiomyocytes using the well-established hanging drop protocol (Figure 6a; adapted from Boheler, Czyz et al. [33]). Embryoid bodies (EBs) were generated from either Cre^+^ or Cre^−^ CAG-floxSTOP-Cx43-P2A-mCherry G4 mouse ES cells (Figure 6a,b). Western blot analysis and immunofluorescence stainings proved that P2A, mCherry, and Cx43 are overexpressed only in Cre^+^ EBs (Figure 6c,d, middle panel, Supplementary Figure S7a). Furthermore, immunostaining against the muscle-specific marker cardiac α-actinin indicated a similar degree of cardiomyocyte differentiation, suggesting that cardiomyocyte development and differentiation was not greatly altered by Cx43 overexpression (Figure 6d, left panel). This was supported by the finding that both mCherry^+^ (Cre^+^) and mCherry^−^ (Cre^−^) EBs started to beat spontaneously at days 10–12 of differentiation (Supplementary Videos S8 and S9), underscoring the viability of the cardiomyocytes despite Cx43 overexpression. This was further investigated by performing immunostainings with the apoptosis marker cleaved caspase 3. Apoptotic areas were preferentially detected, as expected, in central areas of Cre^+^, as well as Cre^−^ EBs, but there was no obvious difference in apoptosis rates in transgenic and control EBs (Figure 6d, right panel; n = 5). We next wondered whether Cx43 overexpression resulted in functional differences and therefore measured spontaneous beating rates in EB-derived cell clusters using microscopy-based video recordings. Our measurements revealed that the Cre^+^ cell clusters displayed significantly higher beating rates (beating frequency = 75.9 bpm; n = 16) than did Cre^−^ controls (beating frequency = 60.3 bpm; n = 19; unpaired *t*-test: *p* < 0.0001) (Figure 7a; Supplementary Videos S10 and S11). In addition, we also found that the Cre^+^ cell clusters were beating more regularly than the Cre^−^ controls, as their beating rates at 1 Hz over a 5 min period yielded a standard deviation (SD) of 1.9 vs. 5.3, respectively (unpaired *t*-test: *p* < 0.0001; n(Cre^+^) = 16, n(Cre^−^) = 19) (Figure 7a). We therefore wondered whether this could be due to changes in the expression of cardiac Cx isoforms. RT qPCR analysis of Cre^+^ and Cre^−^ undifferentiated ES (data not shown) and EB-derived cells revealed expression of Cx43 and Cx45, but not of Cx30.2 and Cx40 (Supplementary Figure S5a). Besides the expected upregulation of total (endogenous + exogenous) Cx43 in Cre^+^ EBs (unpaired *t*-test: *p* = 0.0028; n = 4), there were no prominent changes in the other Cx isoforms (Supplementary Figure S5a). Thus, these experiments proved intact in vitro differentiation characteristics of Cre^+^ cardiomyocytes despite Cx43 overexpression.

### 3.4. Establishment of a Virus-Based Cx43 Overexpression System

Viruses are powerful tools for the targeting of cells and tissues. We wanted to have another tool for Cx43 overexpression in vitro and in vivo and have therefore generated an AAV2.6-Cx43-P2A-mCherry virus. This harbored the identical expression cassette, which was used above (Supplementary Figure S6a) under direct control by the CAG-promoter. We have chosen the AAV2.6 type, as this serotype is known to transduce not only muscle cells, but also other cell types, such as those of the central nervous system [45]. We tested the feasibility and utility of this virus in neonatal mouse cardiomyocytes (NNCMs), which are frequently used as an in vitro model because adult cardiomyocytes dedifferentiate rapidly in culture and therefore cannot be used for this type of experiment [46]. Enriched NNCMs (Figure 7b) were treated with the virus for two days and three days post-transduction; mCherry^+^ NNCMs were observed (Figure 7c, upper picture). The transduction efficiency of the AAV2.6-Cx43-P2A-mCherry virus amounted to 47.7% in NNCMs (n = 3, 531 mCherry^+^ NNCMs out of a total of 1113 NNCMs) (Figure 7c, graph). Cell clusters were observed to beat spontaneously before and after viral transduction, underscoring the viability of NNCMs (Supplementary Videos S12 and S13). Immunofluorescence stainings confirmed P2A-tagged Cx43 overexpression in mCherry^+^ NNCMs (Figure 7d, upper panel). Cellular distribution of Cx43 was mainly restricted to the membrane in both control (Figure 7d, second and fourth panel) and transduced cardiomyocytes (Figure 7d, upper and third panel); to a small degree, cytosolic localization was also detected. Positive cardiac α-actinin staining of mCherry^+^ cells corroborated the successful viral targeting of NNCMs (Figure 7d, third panel). These experiments demonstrated the successful development of a virus-based transduction system that allows targeted overexpression of Cx43, as well as mCherry and P2A labelling.

## 4. Discussion

Herein we report the generation of a new genetic model that allows inducible overexpression of Cx43 in mouse ES cells. Cx43 is widely distributed in mammals, and we have chosen the ES cell system because in vitro differentiation of pluripotent cells allows us to assess the effects of Cx43 overexpression in different somatic cell types, such as cardiomyocytes and astrocytes. We have also established an AAV virus, which enables Cx43 overexpression of cells, tissues, and organs in vitro and in vivo.

We have opted for an inducible genetic system because CMV-driven ubiquitous Cx43 overexpression in mice was reported to cause morbidity and postnatal mortality due to neural tube and conotruncal heart defects [29], a phenotype which was reminiscent of Cx43 KO models [21,47]. We tested our genetic construct in ES cells, as well as HeLa cells and 3T3-fibroblasts, and observed no leakiness in the absence of Cre, whereas AAV-Cre-mediated excision of the floxed stop cassettes resulted in Cx43 overexpression in cells with and without endogenous Cx43 expression. A double reporter system was implemented to detect both Cx43 overexpressing cells based on mCherry fluorescence and the location and distribution pattern of exogenous Cx43 molecules based on their fusion to the small self-cleaving peptide P2A [48], which is detectable by antibodies (Figure 2c,d). P2A is a powerful tool, as it allows co-translational expression of P2A-linked genes in equal amounts in contrast to IRES sites [49]. Earlier work reported that the tagging of Cx43 is challenging, as fusion of proteins to its N-terminus resulted in the expression of non-functional hemichannels and gap junctions [43], fusion to its C-terminus in altered gating properties [50], and altered regulation of connexons and gap junctions [44,51]. We therefore chose to fuse the small P2A peptide to the C-terminal site of Cx43, and our data clearly show that this did neither alter the expression pattern nor the biology of the exogenous protein. This was underscored by dye diffusion in HeLa cells and FRAP experiments in 3T3-fibroblasts and G4 ES cells, proving the formation of functional Cx43 gap junction channels. As perturbation of the C terminus of Cx43 could lead to alterations of interactions with proteins involved in plaque dynamics [52], this aspect needs be explored in the future by performing specific biochemical assays [53,54]. Similarly, phosphorylation is known to be a key modulator of Cx43 gap junction function, and, therefore, the phosphorylation pattern of exogeneous Cx43 should be investigated using phospho-specific antibodies [55]. When analyzing the exogeneous Cx43 distribution pattern with confocal microscopy, we also found that it overlapped with endogenous Cx43 (Figure 2c, upper panel; Figure 4a, upper panel). Super-resolution microscopy and biochemical approaches are required to determine whether exogenous P2A^+^- and endogenous P2A^−^-Cx43 molecules assemble to form connexons and functional gap junctions.

Because of the reported adverse effects of Cx43 overexpression in vivo, we have explored this aspect in our system and differentiated mouse ES cells into spontaneously beating EBs in vitro upon Cre-induction. We could not observe prominent cell biological differences, as neither cardiac α-actinin immunostainings (Figure 6d, left panel), nor the degree of apoptosis (Figure 6d, right panel), nor the time point of initiation of spontaneous beating differed strongly between Cx43 overexpression and control EBs. Interestingly, when quantifying beating rates in Cx43 overexpression and control cell clusters, we noticed that mutant clusters were beating at faster rates and in a clearly more rhythmic fashion. At this point, we can only speculate on the mechanism and propose that the ubiquitous Cx43 overexpression provides better overall conduction between cardiomyocytes and also with non-cardiomyocytes within EBs, as we did not detect changes in the expression of other cardiac Cx isoforms. Recent experiments in human-induced pluripotent cell-derived cardiac microtissues found that Cx43 gap junctions are critical for the maturation of cardiomyocytes in these constructs and that this depends on the diffusion of cAMP through Cx43 gap junction channels [56]. It is possible that also, in our case, the observed higher beating rates are related to more mature cardiomyocytes, which is difficult to assess based on purely morphological criteria. As expected for cultured cardiomyocytes, endogenous and exogenous Cx43 was found to be distributed in ES cell-derived cardiomyocytes and in NNCMs diffusely on the cell surface, whereas in the mature heart, its location is primarily restricted to intercalated discs.

Taken together, we have generated and characterized an inducible Cx43 overexpression system in murine ES cells and an AAV-Cx43 overexpression system enabling the investigation of both the cell biological and functional consequences of overexpressing Cx43 in vitro and in vivo. We envision for both systems multiple applications. Our group could show that grafting of Cx43-expressing muscle cells into the infarct area or overexpression of Cx43 in resident myofibroblasts of the scar strongly reduced the incidence of post-infarction ventricular tachycardias in mice in vivo [37,57]. Despite the great translational potential of these strategies for specific patient cohorts, potential cell biological (e.g., cell differentiation, maturation, viability) and functional (e.g., expression of ion channels and transporters) drawbacks of Cx43 (over)expression in cardiomyocytes and non-cardiomyocytes need to be investigated. The in vitro ES cell differentiation system is ideally suited for this purpose. Similarly, it has been suggested that some forms of epilepsy could benefit from the increased opening of Cx43 gap junction channels in astrocytes [58]. Therefore, novel Cx43 gap junction activators and openers could be studied in ES cell-derived astrocytes in regard to their effects on Cx43 gap junction channels. In addition, potential adverse effects of such Cx43 modulators could be investigated in terms of safety pharmacology in transgenic ES cell-derived cardiomyocytes. The transgenic G4 ES cells containing the CAG-floxSTOP-Cx43-P2A-mCherry construct can be used to generate transgenic mice. Such a mouse model would allow for the testing of the therapeutic potential of Cx43 overexpression in any cell type, depending on crossings with Cre-deleter mouse lines. For instance, for us and other groups working on cardiac arrhythmias, it would be of great interest to induce homogenous overexpression of Cx43 in border zone cardiomyocytes or myofibroblasts of the scar area and to study the effects on ventricular arrhythmias after myocardial infarction.

## Data Availability

Not applicable.

## Supplementary Materials

The following supporting information can be downloaded at: https://www.mdpi.com/article/10.3390/cells11040694/s1: Figure S1: Identification of suitable transgenic cell clones after transfection of the expression plasmid and subsequent selection in murine G4 ES cells, Figure S2: Dye diffusion experiments in HeLa cells using the large MW dye Alexa647-dextran, Figure S3: Generation of Cre^+^ 3T3-fibroblast lines, fluorescence recovery after photobleaching (FRAP) experiment, Figure S4: Fluorescence recovery after photobleaching (FRAP) experiments in G4 ES cells, Figure S5: Expression of different Cx isoforms in embryoid bodies (EBs) derived from transgenic Cre^+^ and Cre^−^ G4 ES cells of clone 31, Figure S6: AAV2.6-CAG-Cx43-P2A-mCherry virus, Figure S7: Western blot of undifferentiated G4 ES cells and EBs using a Cx43 antibody (Sigma-Aldrich, Merck, #C6219), Video S1: FRAP experiments of Cre^+^ 3T3-fibroblasts_Calcein-AM, Video S2: FRAP experiments of Cre^+^ 3T3-fibroblasts_mCherry, Video S3: FRAP experiments of Cre^−^ 3T3-fibroblasts_Calcein-AM, Video S4: FRAP experiments of WT 3T3-fibroblasts_Calcein-AM, Video S5: FRAP experiments of Cre^+^ G4 ES cell_Calcein-AM, Video S6: FRAP experiments of Cre^+^ G4 ES cell_mCherry, Video S7: FRAP experiments of Cre^−^ G4 ES cell_Calcein-AM, Video S8: Beating Cre^+^ EB, Video S9: Beating Cre^−^ EB, Video S10: Beating Cre^+^ cell cluster after partial dissociation of EBs and plating, Video S11: Beating Cre^−^ cell cluster after partial dissociation of EBs and plating, Video S12: Beating monolayer of neonatal cardiomyocytes (NNCMs) (P1) one day post isolation, Video S13: Beating monolayer of NNCMs (P1) three days post transduction with AAV2.6-Cx43-P2A-mCherry virus.
