# Supplementary material for: Generation and Characterization of an Inducible Cx43 Overexpression System in Mouse Embryonic Stem Cells
Source: Cells. 2022 Feb 16;11(4):694. doi: 10.3390/cells11040694 (PMC8869955; doi:10.3390/cells11040694)
Supplement: Supplementary file 1 [file cells-11-00694-s001.zip › cells-1543381 supplementary.pdf]

cells

Article

Generation and characterization of an inducible Cx43 overexpression system in mouse embryonic stem cells

Pia Niemann [1], Miriam Schiffer [1,2], Daniela Malan [1], Sabine Grünberg [1], Wilhelm Roell [2], Caroline Geisen [1,2,*], and Bernd K. Fleischmann [1,*]

[1] Institute of Physiology I, Life and Brain Center, Medical Faculty, University of Bonn, Bonn, 53127, Germany
[2] Department of Cardiac Surgery, University Hospital Bonn, Bonn, 53127, Germany
* Correspondence: CG: cgeisen@uni-bonn.de; BKF: bernd.fleischmann@uni-bonn.de, Tel.: (+49) 0228 6885 200

3.2. Supplementary Figures

Citation: Niemann, P.; Schiffer, M.; Malan, D.; Grünberg, S.; Roell, W.; Geisen, C.; Fleischmann, B.K. Generation and Characterization of an Inducible Cx43 Overexpression System in Mouse Embryonic Stem Cells. *Cells* **2022**, *11*, 694. https://doi.org/10.3390/cells11040694

Academic Editors: Sebastian Clauß and Ling Xiao

Received: 21 December 2021
Accepted: 9 February 2022
Published: 16 February 2022

Publisher's Note: MDPI stays neutral with regard to jurisdictional claims in published maps and institutional affiliations.

Copyright: © 2022 by the authors. Licensee MDPI, Basel, Switzerland. This article is an open access article distributed under the terms and conditions of the Creative Commons Attribution (CC BY) license (https://creativecommons.org/licenses/by/4.0/).

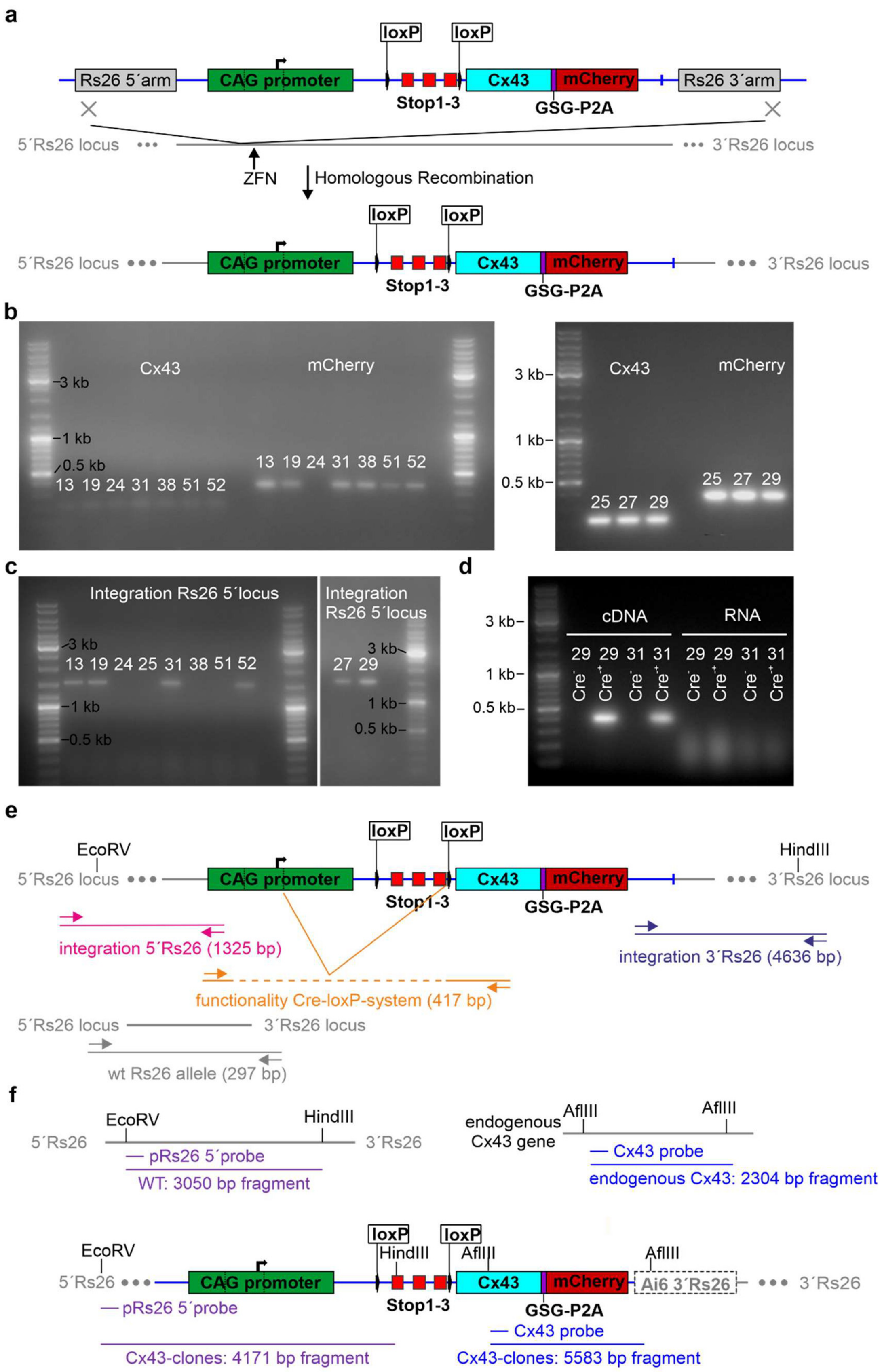

Figure S1. Identification of suitable transgenic cell clones after transfection of the expression plasmid and subsequent selection in murine G4 ES cells. (**a**) Scheme for homologous integration of the transgene into the endogenous Rs26 locus of G4 mouse ES cells after co-transfection of the CAG-floxSTOP-Cx43-P2A-mCherry-Ai6

expression vector and zinc finger nucleases (ZFN) into murine G4 ES cells. (**b**) PCR analysis of ES cell clones proved the presence of Cx43 (band: 213 bp) and mCherry (band: 378 bp) DNA. (**c**) It also showed for the 5′ end correct integration of the transgene into the Rs26 locus (band: 1325 bp). (**d**) RT-PCR analysis performed prior (Cre$^-$) and after Cre induction via AAV2.1-Cre transduction and selection of single subclones (Cre$^+$) underscored intact function of the Cre-loxP system in ES cell clones 29 and 31 (band: 417 bp). (**e**) Scheme for primer pairs of PCR and RT-PCR analysis. Expected band sizes for integration of the transgene into the 5′-side (1325 bp) and 3′-side (4636 bp) of the Rs26 locus, and for the functionality of the Cre-loxP system (417 bp) are given (upper panel). Binding of the Rs26 specific primer pair would result in a 297 bp band, in case one WT Rs26 locus was intact (lower panel). (**f**) Scheme for the binding of Southern blot probes, DNA was double digested with EcoRV and HindIII or with AflIII alone. A flanking (pRs26-5′) and a Cx43 probe were used. In case of the WT allele (upper panel) the pRs26-5' probe detected the 3050 bp fragment of the Rs26 locus, and the Cx43 probe hybridized to the endogenous Cx43 fragment (2304bp). Correct integration of the insert into the Rs26 locus (lower panel) resulted in a 4171 bp fragment after hybridization with the pRs26-5' probe and yielded an additional 5583 bp fragment with the Cx43 probe.

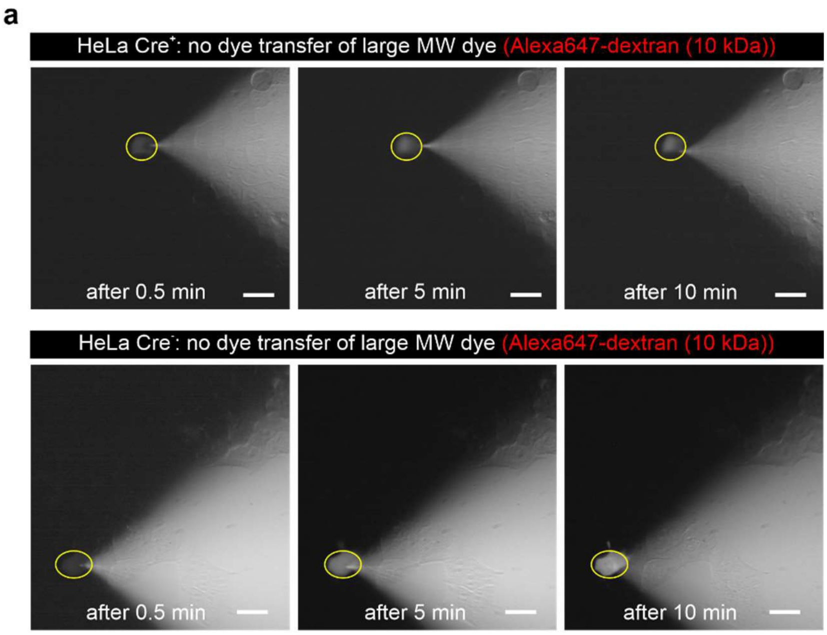

Figure S2. Dye diffusion experiments in HeLa cells using the large MW dye Alexa647-dextran. (**a**) Upon dialysis of a single HeLa cell with the large MW dye Alexa647-dextran neither in Cre$^+$ (upper panel) nor in Cre$^-$ (lower panel) HeLa cells dye diffusion into adjacent cells was observed, ruling out cytoplasmatic bridge formation. Scale bars 100 µm. Dialyzed cell marked by a yellow circle.

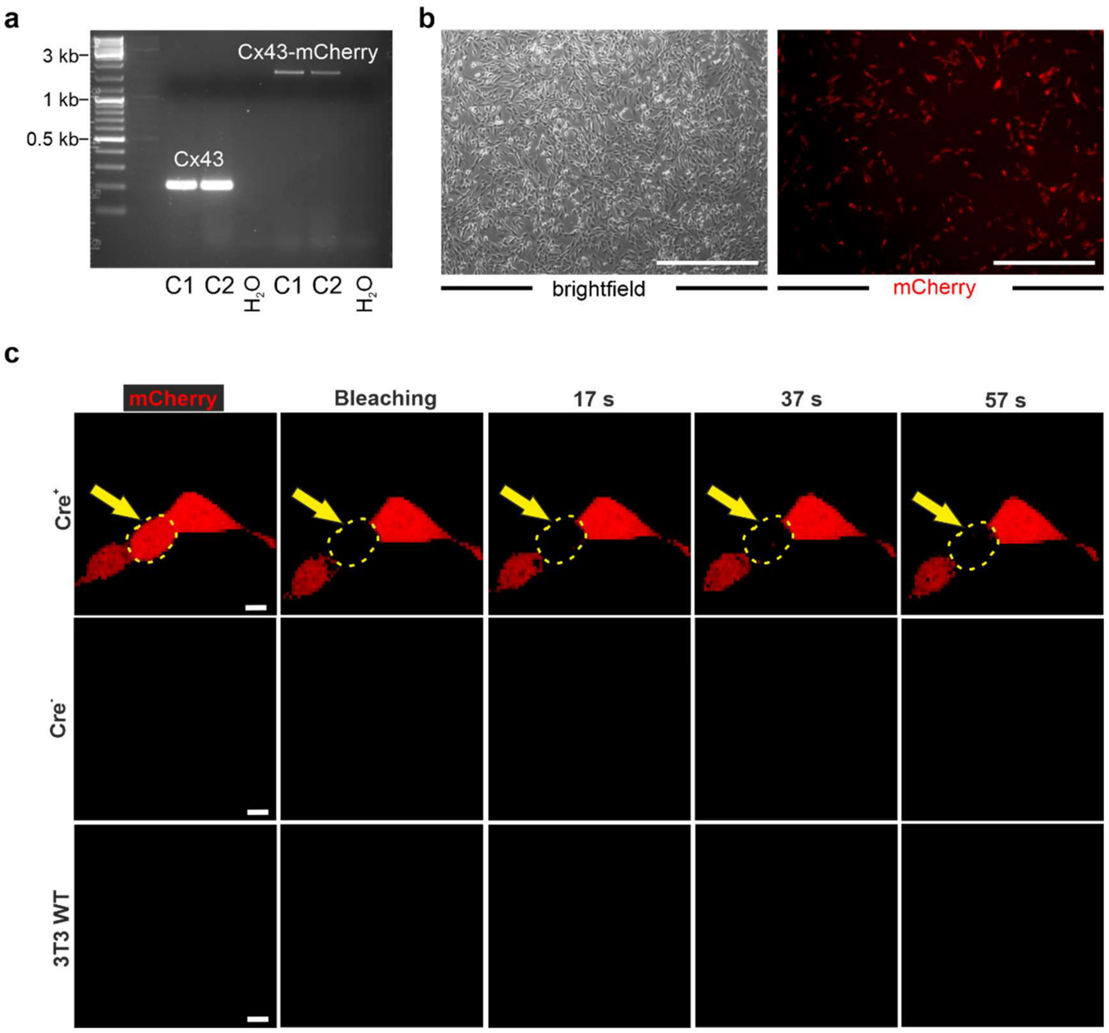

Figure S3. Generation of Cre+ 3T3-fibroblast lines, fluorescence recovery after photobleaching (FRAP) experiments. (**a**) PCR analysis with primers for Cx43 (left side, 213 bp) as well as Cx43 and mCherry (right side, 1658 bp) proved successful integration of the transgene in 3T3-fibroblasts of clones 1 and 2. (**b**) Transgenic Cre+ 3T3-fibroblasts showed mCherry expression 3 days after AAV2.1-Cre transduction. Scale bar 100 µm. (**c**) Original fluorescence pictures of 3T3 Cre+, Cre- and WT fibroblasts, respectively, after calcein AM (0.38 µM) (Figure 4d) dye loading, bleaching (for 5 s with a 561 nm laser with 2.5 mW intensity) of a single fibroblast (bleached cell marked by a yellow dotted line and a yellow arrow) and dye recovery at 17 s, 37 s and 57 s after bleaching; mCherry (red). Scale bars 20 µm.

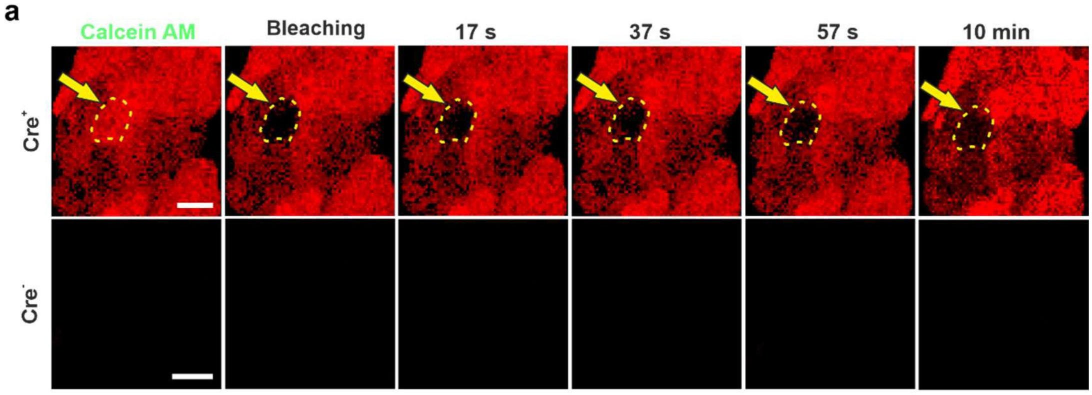

Figure S4. Fluorescence recovery after photobleaching (FRAP) experiments in G4 ES cells. (**a**) Original fluorescence pictures of Cre⁺ and Cre⁻ G4 ES cells after Calcein AM (0.38 µM) (Figure 5a) dye loading, bleaching (for 5 s with a 561 nm laser with 2.5 mW intensity) of a single G4 ES cell of the cell cluster (bleached cell marked by a yellow dotted line and a yellow arrow) and dye recovery at 17 s, 37 s, 57 s and 10 min after bleaching; mCherry (red). Scale bars 20 µm.

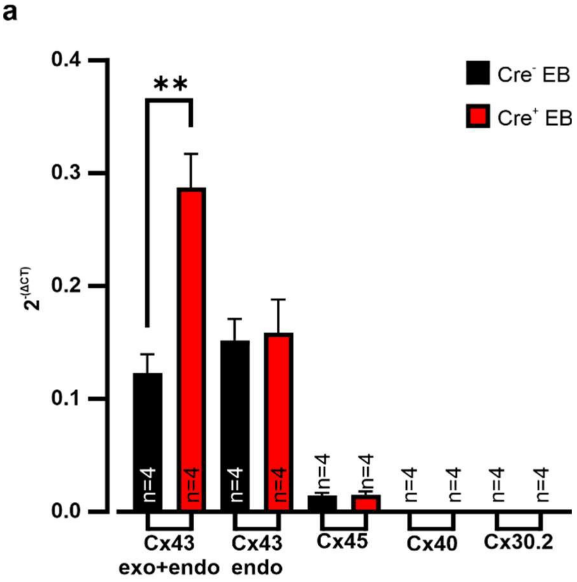

Figure S5. Expression of different Cx isoforms in embryoid bodies (EBs) derived from transgenic Cre⁺ and Cre⁻ G4 ES cells of clone 31. (**a**) RT qPCR analysis showed a significantly increased total (exogenous + endogenous) Cx43 expression in Cre⁺ EBs compared to Cre⁻ controls, whereas endogenous Cx43 was found to be not differently expressed. Cx45 expression was similar in Cre⁺ and Cre⁻ EBs. Other Cx isoforms (Cx40 and Cx30.2) were not detectable. GAPDH was used as housekeeping gene. ** p value ≤ 0.01.

a

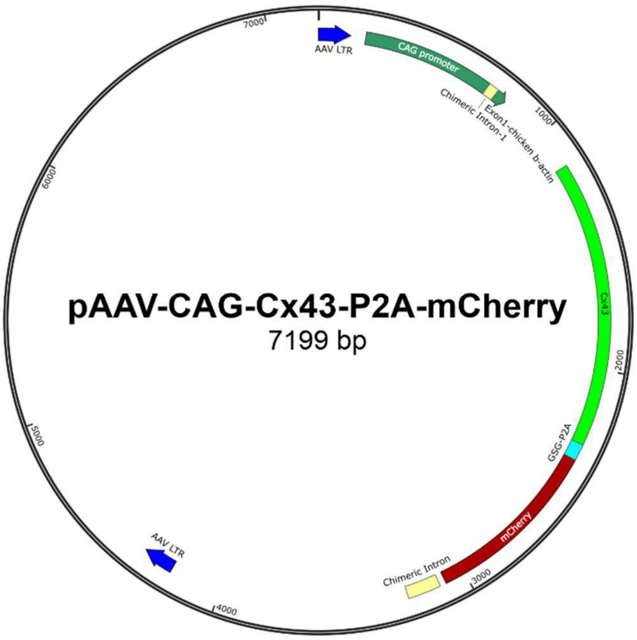

Figure S6. AAV2.6-CAG-Cx43-P2A-mCherry virus. (**a**) Scheme of the AAV2.6-CAG-Cx43-P2A-mCherry virus vector.

a

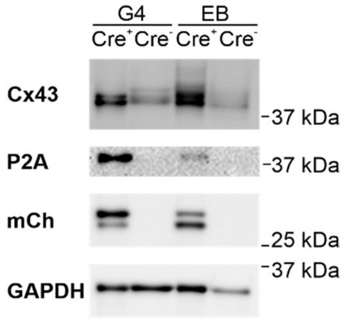

Figure S7. Western blot of undifferentiated G4 ES cells and EBs using a Cx43 antibody (Sigma Aldrich #C6219). (**a**) Western blot analysis of undifferentiated and differentiated (EBs) Cre+ and Cre- G4 ES cells of C31 proved inducible Cx43 overexpression. Only AAV2.1-Cre treated cells showed mCherry- and P2A expression.

Video S1. FRAP experiments of Cre+ 3T3-fibroblasts. Upon loading with calcein AM dye, a single 3T3-fibroblast was bleached with a 561 nm laser, fluorescence recovery was monitored over time. In Cre+ Cx43 overexpressing 3T3 cells fast fluorescence recovery after photobleaching (FRAP) was observed. Calcein (green).

Video S2. FRAP experiments of Cre+ 3T3-fibroblasts. A single 3T3-fibroblast was bleached with a 561 nm laser, fluorescence recovery was monitored over time. mCherry fluorescence (red) was not recovered.

Video S3. FRAP experiments of Cre- 3T3-fibroblasts. Upon loading with calcein AM dye, a single Cre- 3T3-fibroblast was bleached with a 561 nm laser, fluorescence recovery was monitored over time. In Cre- 3T3-fibroblasts slow FRAP was observed. Calcein (green).

Video S4. FRAP experiments of WT 3T3-fibroblasts. Upon loading with calcein AM dye a single WT 3T3-fibroblast was bleached with a 561 nm laser, fluorescence recovery was monitored over time. In WT 3T3-fibroblasts only slow FRAP was observed. Calcein (green).

Video S5. FRAP experiments of Cre^{+} G4 ES cells. Upon loading with calcein AM dye, a single G4 ES cell was bleached with a 561 nm laser, fluorescence recovery was monitored over time. In Cre^{+} Cx43 overexpressing G4 ES cells fast fluorescence recovery in the initial stages after photobleaching was observed. Calcein AM (green).

Video S6. FRAP experiments of Cre^{+} G4 ES cells. A single G4 ES cell was bleached with a 561 nm laser, fluorescence recovery was monitored over time. mCherry fluorescence (red) was not recovered.

Video S7. FRAP experiments of Cre^{-} G4 ES cells. Upon loading with calcein AM dye, a single Cre^{-} G4 ES cell was bleached with a 561 nm laser, fluorescence recovery was monitored over time. In Cre^{-} G4 ES cells FRAP was found to be slower in the initial stages after bleaching. Calcein AM (green).

Video S8. Beating Cre^{+} EB. Spontaneously beating Cre^{+} EB at d 12 of differentiation.

Video S9. Beating Cre^{-} EB. Spontaneously beating Cre^{-} EB at d 12 of differentiation.

Video S10. Beating Cre^{+} cell cluster after partial dissociation of EBs and plating. Spontaneously beating Cre^{+} Cx43 overexpressing cell clusters one day after dissociation of EBs exhibited higher spontaneous beating rates and more regular beating compared to controls.

Video S11. Beating Cre^{-} cell cluster after partial dissociation of EBs and plating. Spontaneously beating Cre^{-} cell clusters one day after dissociation of EBs exhibited lower beating rates and more irregular beating compared to Cre^{+} cell clusters.

Video S12. Beating monolayer of neonatal cardiomyocytes (NNCMs) (P1) one day post isolation. Spontaneous beating of a monolayer of NNCMs.

Video S13. Beating monolayer of NNCMs (P1) three days post transduction with AAV2.6-Cx43-P2A-mCherry virus. Spontaneous beating of a monolayer of $mCherry^{+}$ NNCM (P1) confirming viability of transduced cardiomyocytes
